# A membrane-depolarizing toxin substrate of the *Staphylococcus aureus* type VII secretion system mediates intraspecies competition

**DOI:** 10.1073/pnas.2006110117

**Published:** 2020-08-07

**Authors:** Fatima R. Ulhuq, Margarida C. Gomes, Gina M. Duggan, Manman Guo, Chriselle Mendonca, Grant Buchanan, James D. Chalmers, Zhenping Cao, Holger Kneuper, Sarah Murdoch, Sarah Thomson, Henrik Strahl, Matthias Trost, Serge Mostowy, Tracy Palmer

**Affiliations:** ^a^Centre for Bacterial Cell Biology, Newcastle University Biosciences Institute, Newcastle University, NE2 4HH Newcastle upon Tyne, United Kingdom;; ^b^Section of Microbiology, Medical Research Council Centre for Molecular Bacteriology and Infection, Imperial College London, SW7 2AZ London, United Kingdom;; ^c^Department of Infection Biology, London School of Hygiene & Tropical Medicine, WC1E 7HT London, United Kingdom;; ^d^Medical Research Council Protein Phosphorylation and Ubiquitylation Unit, School of Life Sciences, University of Dundee, DD1 5EH Dundee, United Kingdom;; ^e^Division of Molecular Microbiology, School of Life Sciences, University of Dundee, DD1 5EH Dundee, United Kingdom;; ^f^Division of Molecular and Clinical Medicine, University of Dundee, DD1 9SY Dundee, United Kingdom;; ^g^Biological Services, School of Life Sciences, University of Dundee, DD1 5EH Dundee, United Kingdom;; ^h^Newcastle University Biosciences Institute, Newcastle University, NE2 4HH Newcastle upon Tyne, United Kingdom

**Keywords:** type VII secretion system, *Staphylococcus aureus*, zebrafish, membrane-depolarizing toxin, bacterial competition

## Abstract

The type VII protein secretion system (T7SS) is conserved across *Staphylococcus aureus* strains and plays important roles in virulence and interbacterial competition. To date, only one T7SS substrate protein, encoded in a subset of *S. aureus* genomes, has been functionally characterized. Here, using an unbiased proteomic approach, we identify TspA as a further T7SS substrate. TspA is encoded distantly from the T7SS gene cluster and is found across all *S. aureus* strains as well as in *Listeria* and Enterococci. Heterologous expression of TspA from *S. aureus* strain RN6390 indicates its C-terminal domain is toxic when targeted to the *Escherichia coli* periplasm and that it depolarizes the cytoplasmic membrane. The membrane-depolarizing activity is alleviated by coproduction of the membrane-bound TsaI immunity protein, which is encoded adjacent to *tspA* on the *S. aureus* chromosome. Using a zebrafish hindbrain ventricle infection model, we demonstrate that the T7SS of strain RN6390 promotes bacterial replication in vivo, and deletion of *tspA* leads to increased bacterial clearance. The toxin domain of TspA is highly polymorphic and *S. aureus* strains encode multiple *tsaI* homologs at the *tspA* locus, suggestive of additional roles in intraspecies competition. In agreement, we demonstrate TspA-dependent growth inhibition of RN6390 by strain COL in the zebrafish infection model that is alleviated by the presence of TsaI homologs.

## Significance

*Staphylococcus aureus*, a human commensal organism that asymptomatically colonizes the nares, is capable of causing serious disease following breach of the mucosal barrier. *S. aureus* strains encode a type VII secretion system that is required for virulence in mouse infection models, and some strains also secrete a nuclease toxin by this route that has antibacterial activity. Here we identify TspA, widely found in Staphylococci and other pathogenic bacteria, as a type VII substrate. We show that TspA has membrane-depolarizing activity and that *S. aureus* uses TspA to inhibit the growth of a bacterial competitor in vivo.

The type VII secretion system (T7SS) has been characterized in bacteria of the actinobacteria and firmicutes phyla. In pathogenic mycobacteria, the ESX-1 T7SS secretes numerous proteins that are essential for virulence and immune evasion (1). The Ess T7SS of *Staphylococcus aureus* is also required for pathogenesis in murine models of infection (2–4), and a longitudinal study of persistent *S. aureus* infection in the airways of a cystic fibrosis patient showed that the *ess* T7SS genes were highly up-regulated during a 13-y timespan (5). It is becoming increasingly apparent, however, that in addition to having antieukaryotic activity, the T7SS of firmicutes mediates interbacterial competition (6–8). Some strains of *S. aureus* secrete a DNA endonuclease toxin, EsaD (6, 9), that when overproduced leads to growth inhibition of a sensitive *S. aureus* strain (6). Moreover, *Streptococcus intermedius* exports at least three LXG domain-containing toxins—TelA, TelB, and TelC—that mediate contact-dependent growth inhibition against a range of gram-positive species (7).

**Fig. 1.**
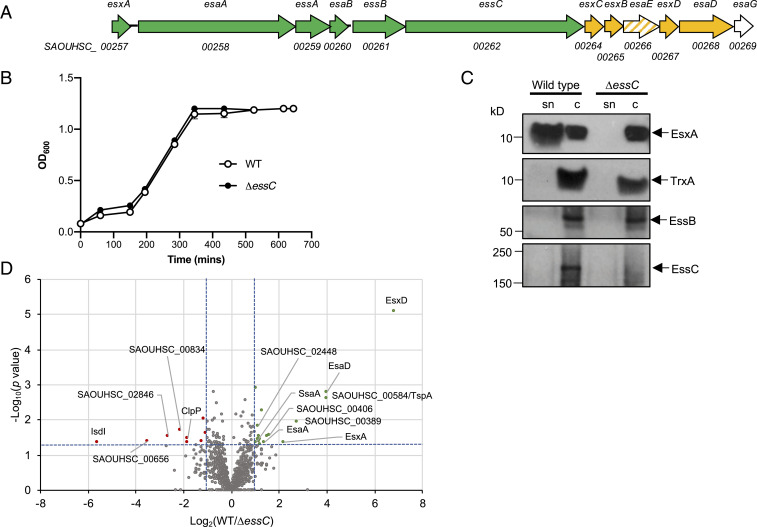
The *S. aureus* RN6390 T7 secretome. (*A*) The *ess* locus in strain NCTC8325 (parent of RN6390). Genes for core components are shaded green, secreted substrates yellow, EsaE (which is cosecreted with EsaD) in hatched shading, and the cytoplasmic antitoxin EsaG in white. (*B*) Growth of RN6390 (WT) and the isogenic Δ*essC* strain in RPMI medium. Points show mean ± SEM (*n* = 3 biological replicates). (*C*) RN6390 (WT) and the Δ*essC* strain cultured in RPMI growth medium to OD_600_ = 1. Samples were separated into supernatant (sn) and cellular (c) fractions (12% Bis-Tris gels) and immunoblotted with anti-EsxA, anti-EssB, anti-EssC, or anti-TrxA (cytoplasmic protein) antisera. (*D*) Volcano plot of the quantitative proteomic secretome analysis. Each spot represents an individual protein ranked according to its statistical *P* value (*y* axis) and relative abundance ratio (log_2_ fold change). The blue dotted lines represent cutoffs for significance (*P* < 0.05; log_2_ fold-change > 1).

A large integral membrane ATPase of the FtsK/SpoIIIE family, termed EssC in firmicutes, is a conserved component of all T7SSs and probably energizes protein secretion as well as forming part of the translocation channel (10–15). EsxA, a small secreted protein of the WXG100 family, is a further conserved T7 component that is dependent on the T7SS for its translocation across the membrane (2, 16). In firmicutes, three additional membrane proteins—EsaA, EssA, and EssB—function alongside the EssC ATPase to mediate T7 protein secretion (17, 18). In *S. aureus* the T7 structural components are encoded at the *ess* locus. In commonly studied strains including Newman, RN6390, and USA300, the T7 substrates EsxB, EsxC, EsxD, and EsaD are encoded immediately downstream of *essC* (Fig. 1*A*) and are coregulated with the genes coding for machinery components (2, 3, 6, 9, 19, 20). With the exception of EsaD, the biological activities of these substrates are unknown, although mutational studies have suggested that EsxB and EsxC contribute to persistent infection in a murine abscess model (2, 19).

Despite the *ess* locus forming part of the core *S. aureus* genome, these four substrate proteins are not conserved across *S. aureus* isolates, being found in only ∼50% of sequenced strains (21). Furthermore, inactivation of the T7SS in *S. aureus* strain ST398 shows a similar decrease in kidney abscess formation as that seen for T7 mutants in Newman and USA300 (2, 4, 22), despite the fact that recognizable homologs of EsxB, EsxC, EsxD, and EsaD are not encoded by this strain (21). This strongly suggests that there are further *S. aureus* T7 substrates that are yet to be identified. Here we have taken an unbiased approach to identify T7 substrates using quantitative proteomic analysis of culture supernatants from *S. aureus* RN6390 wild-type and *essC* strains. We identify a substrate, TspA, that is encoded distantly from the *ess* gene cluster and is found in all sequenced *S. aureus* strains. Further analysis indicates that TspA has a toxic C-terminal domain that depolarizes membranes. Using a zebrafish hindbrain ventricle infection model, we reveal that the T7SS and TspA contribute to both bacterial replication and interbacterial competition in vivo.

## Results

### The *S. aureus* RN6390 T7SS Secreted Proteome.

To identify candidate T7 substrates, RN6390 and an isogenic *essC* deletion strain (3) were cultured in the minimal medium RPMI. Both strains grew identically (Fig. 1*B*) and expressed components of the T7SS, and as expected secretion of EsxA was abolished in the *essC* strain (Fig. 1*C*). Culture supernatants were isolated when cells reached OD_600nm_ of 1, and label-free quantitative proteomics was used to assess changes in protein abundance of four biological replicates of each secretome. Following identification of 1,170 proteins, 17 proteins showed, with high confidence (*P* < 0.05, two fold change), a decrease in abundance in the secretome of the *essC* strain relative to the RN6390 parent strain (Fig. 1*D*, Table 1, and Dataset S1).

**Table 1.** Proteins present in the secretome of RN6390 at an abundance of greater than twofold higher than the secretome of the isogenic Δ*essC* strain

Identifier	Protein name/description	Relative abundance WT/Δ*essC*	*P* value	Unique peptides	Sequence coverage, %
SAOUHSC_00267	EsxD (T7 secreted substrate)	113.6*	8.00 × 10^−6^	4	47.6
SAOUHSC_00584	(TspA) Uncharacterized LXG domain protein	15.9	2.45 × 10^−3^	16	44.3
SAOUHSC_00268	EsaD (T7 secreted nuclease)	15.8	1.64 × 10^−3^	24	44.5
SAOUHSC_00389	Uncharacterized. Predicted superantigen-like protein	6.7*	0.00109	2	25.6
SAOUHSC_00257	EsxA (Secreted T7 core component)	4.6	0.00425	6	81.4
SAOUHSC_00406	Uncharacterized protein	3.0	0.00272	10	31.7
SAOUHSC_01342	Nuclease SbcCD subunit C	2.9	0.00291	7	9.7
SAOUHSC_01949	Intracellular serine protease, putative	2.6	0.00421	7	20.8
SAOUHSC_02028	PhiETA ORF57-like protein	2.4	5.21 × 10^−3^	13	26.4
SAOUHSC_01191	50S ribosomal protein L28	2.3	0.00498	3	24.2
SAOUHSC_02695	Uncharacterized protein with DUF4467/cystatin-like domain	2.3	0.00337	5	31
SAOUHSC_01180	Uncharacterized protein	2.3	0.00286	26	73.2
SAOUHSC_00258	EsaA (membrane-bound T7 core component)	2.2	0.00397	71	59.4
SAOUHSC_02448	Uncharacterized protein with alpha/beta hydrolase fold	2.1	0.00353	17	59.2
SAOUHSC_02042	Phi Mu50B-like protein	2.1	0.00141	2	18.9
SAOUHSC_02027	SLT orf 129-like protein	2.1	1.22 × 10^−3^	4	56
SAOUHSC_02883	Staphylococcal secretory antigen SsaA	2.0	0.00431	5	43.1

A full list of all of the proteins identified in this analysis is given in Dataset S1.

* Not detected in the Δ*essC* secretome.

Proteomic analysis indicated that the secreted core component, EsxA, was significantly reduced in abundance in the *essC* secretome, as expected from the Western blot analysis (Fig. 1*C*). Peptides from the membrane-bound T7 component EsaA, which has a large surface-exposed loop (23), were also less abundant in the supernatant of the *essC* strain, as were EsxD and EsaD, known substrates of the T7SS (6, 9, 20) (Table 1 and Dataset S1). The EsxC substrate (19) was also exclusively detected in the supernatant of the wild-type strain, but only two EsxC peptides were detected and it did not meet the *P* < 0.01 cutoff (Dataset S1). EsxB, another previously identified substrate (2, 24), and EsaE, which is cosecreted with EsaD (6), were not detected in any of our analysis.

After EsxD, the protein with the highest relative abundance in the secretome of the wild-type strain was the uncharacterized protein SAOUHSC_00584. This protein harbors a predicted LXG domain, which is common to some T7SS substrates (7). Other proteins enriched in the secretome of the wild-type strain include a predicted superantigen (SAOUHSC_00389), the secretory antigen SsaA, two predicted membrane-bound lipoproteins (SAOUHSC_01180 and SAOUHSC_02695), two uncharacterized proteins (SAOUHSC_00406 and SAOUHSC_02448), and several predicted cytoplasmic proteins (Table 1). A small number of proteins were found to be enriched in abundance in the *essC* secretome (Dataset S1), including the heme oxygenase IsdI, which is known to be up-regulated when the T7SS is inactivated (25).

### SAOUHSC_00584/TspA Localizes to the Membrane Dependent on EssC.

**Fig. 2.**
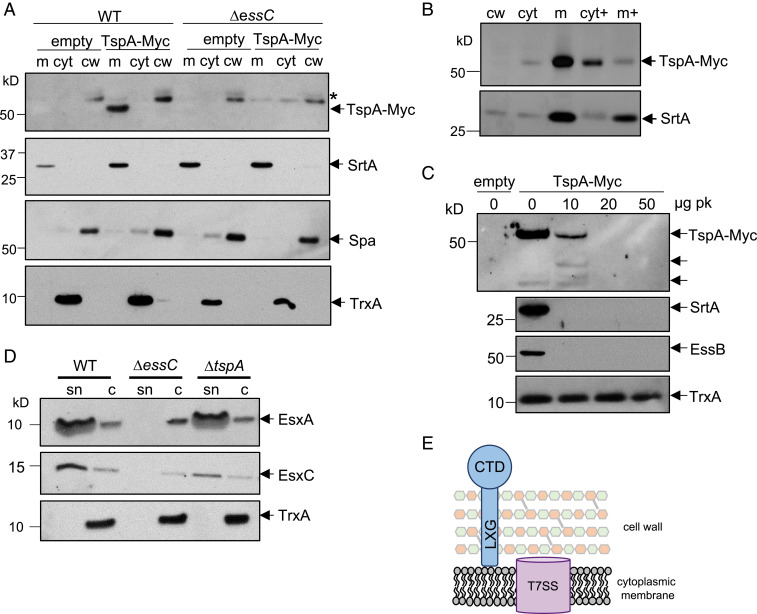
SAOUHSC_00584/TspA is an extracellular peripheral membrane protein. (*A*) RN6390 (WT) and the Δ*essC* strain harboring pRAB11 (empty) or pRAB11-TspA-Myc were cultured in TSB growth medium. Following induction of plasmid-encoded TspA-Myc production, cells were harvested and fractionated into cell wall (cw), membrane (m), and cytoplasmic (cyt) fractions. Samples were separated (12% Bis-Tris gels) and immunoblotted with anti–Myc-HRP, anti-TrxA (cytoplasmic protein), anti-Spa (cell wall), or anti-SrtA (membrane) antisera. An asterisk (*) represents nonspecific cross-reacting band corresponding to Spa. (*B*) Cell extracts from the RN6390 samples in *A* were incubated with 4 M urea, membranes were isolated and the urea-treated cytoplasm (cyt+) and membranes (m+) were separated alongside the cell wall and untreated cytoplasm and membrane fractions on a 12% Bis-Tris gel and immunoblotted with anti-Myc and anti-SrtA antisera. (*C*) Spheroplasts from strain RN6390 producing TspA-Myc were incubated with the indicated concentrations of Proteinase K (pk) at 4 °C for 30 min. A sample of spheroplasts from RN6390 containing pRAB11 (empty) is shown as a negative control. Samples were separated on a 12% Bis-Tris gel and immunoblotted using anti-Myc, anti-SrtA, anti-EssB, and anti-TrxA antisera. (*D*) *S. aureus* RN6390 or the isogenic Δ*essC* or Δ*tspA* strains were cultured in TSB medium and harvested at OD_600_ of 2. Supernatant (sn) and cellular (c) fractions (equivalent of 100 μL culture supernatant and 10 μL of cells adjusted to OD_600_ of 2) were separated on Bis-Tris gels (15% acrylamide) and immunoblotted using anti-EsxA, EsxC, or TrxA antisera. (*E*) Model for organization of TspA in the *S. aureus* envelope. CTD, C-terminal (channel-forming) domain.

We next constructed tagged variants of each of SAOUHSC_00389, SAOUHSC_00406, SAOUHSC_00584, SAOUHSC_02448, and SsaA to probe their subcellular locations in the wild-type and Δ*essC* strains. C-terminally HA-tagged SAOUHSC_00389, SAOUHSC_02448, and SsaA were secreted into the culture supernatant in an *essC*-independent manner (*SI Appendix*, Fig. S1), indicating that these proteins are not substrates of the T7SS and their reduced abundance in the *essC* secretome may arise for pleiotropic reasons. Overproduction of C-terminally Myc-tagged SAOUHSC_00406 caused cell lysis, seen by the presence of TrxA in the supernatant samples (*SI Appendix*, Fig. S1*B*). In contrast, a C-terminally Myc-tagged variant of SAOUHSC_00584 was detected only in the cellular fraction (*SI Appendix*, Fig. S1*C*). To probe the subcellular location of SAOUHSC_00584-Myc, we generated cell wall, membrane, and cytoplasmic fractions. Fig. 2*A* shows that the tagged protein localized to the membrane and that it appears to be destabilized by the loss of EssC. SAOUHSC_00584 was subsequently renamed TspA (type seven dependent protein A).

TspA is predicted to be 469 amino acids long and to have either one (TMHMM) or two (Predictprotein.org) transmembrane domains toward its C-terminal end. To determine whether it is an integral membrane protein, we treated membranes with urea, which removes peripherally bound proteins by denaturation. Fig. 2*B* indicates that a large fraction of TspA-Myc was displaced from the membrane to the cytoplasmic fraction by the addition of urea, whereas a bona fide integral membrane protein, EssB (26–28), was not displaced by this treatment. We conclude that TspA-Myc peripherally interacts with the membrane. This is consistent with findings from the proteomic experiment as peptides along the entire length of TspA were detected in the secretome (*SI Appendix*, Fig. S2).

To determine whether TspA-Myc is exposed at the extracellular side, we prepared spheroplasts and treated them with proteinase K. Fig. 2*C* shows that at low concentrations of proteinase K, TspA-Myc was proteolytically cleaved to release a smaller fragment that also cross-reacted with the anti-Myc antibody. At least part of this smaller fragment must be extracellular as it was also degraded as the protease concentration was increased. An ∼37-kDa C-terminal fragment of TspA-Myc detected natively in the absence of added protease was also extracellular as it was sensitive to digestion by proteinase K. The presence of full-length TspA in the culture supernatant in the proteomic analysis is likely due to surface shedding, a phenomenon that has also been seen for the cell wall-anchored protein A (29). The likely topology of TspA is shown in Fig. 2*E*.

All *S. aureus* T7SS substrate proteins identified to date are found only in a subset of strains, and are linked with specific *essC* subtypes. However, TspA is encoded by all *S. aureus* genomes examined in Warne et al. (21), and is distant from the *ess* locus. This raised the possibility that TspA may be a further secreted core component of the T7 machinery. To examine this, we constructed an in-frame *tspA* deletion in RN6390 and investigated the subcellular location of the T7-secreted component EsxA and the substrate protein EsxC. Fig. 2*D* shows that both EsxA and EsxC are secreted in the absence of TspA. We conclude that TspA is a peripheral membrane protein substrate of the T7SS, whose localization and stability at the extracellular side of the membrane is dependent on EssC, and that it is not a core component of the T7SS.

### TspA has a Toxic C-Terminal Domain with Membrane Depolarizing Activity That Is Neutralized by TsaI.

**Fig. 3.**
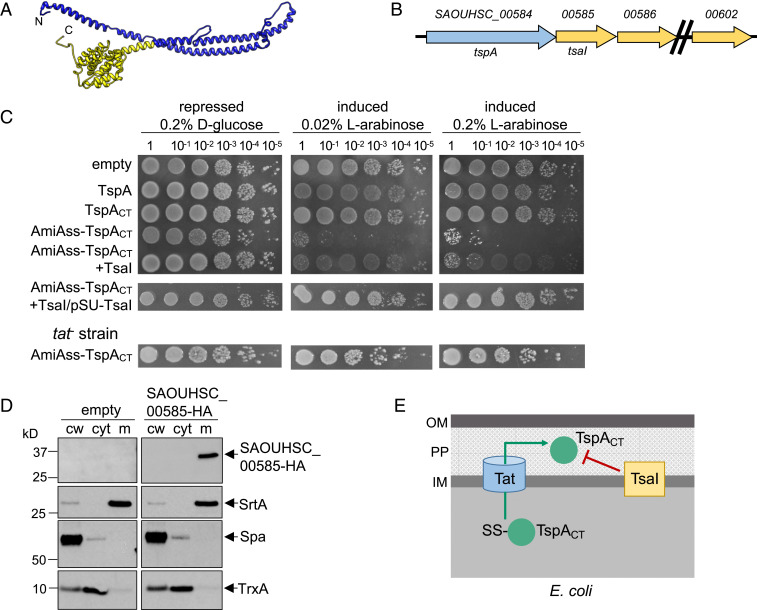
Directed export of TspA C-terminal domain to the periplasm of *E. coli* is toxic. (*A*) Structural model for residues 9 to 416 of TspA generated using RaptorX (raptorx.uchicago.edu/), modeled on the colicin Ia structure (30). The predicted channel-forming region is shown in yellow. (*B*) The *tspA* locus. Genes coding for DUF443 proteins are shown in yellow. (*C*) *E. coli* strain MG1655 harboring empty pBAD18-Cm or pBAD18-Cm encoding either full-length TspA, the TspA C-terminal domain (TspA_CT_), TspA_CT_ with the AmiA signal sequence at its N terminus (AmiAss–TspA_CT_), AmiAss–TspA_CT_ and TsaI (SAOUHSC_00585), AmiAss–TspA_CT_/TsaI alongside an additional plasmid-encoded copy of TsaI (from pSU-TsaI), or strain SG3000 (as MG1655, Δ*tatABCD*) harboring pBAD18-AmiAss–TspA_CT_ was serially diluted and spotted on LB plates containing either d-glucose or l-arabinose, as indicated. Plates were incubated at 37 °C for 16 h, after which they were photographed. (*D*) *S. aureus* cells harboring pRAB11 (empty) and pRAB11-SAOUHSC_00585-HA were cultured in TSB medium and expression of SAOUHSC_00585-HA induced by addition of 500 ng/mL ATc when the cells reached OD_600_ of 0.4. The cells were then harvested at OD_600_ of 2. The cells were spun down and subsequently fractionated into cell wall (cw), membrane (m), and cytoplasmic (cyt) fractions. The fractionated samples were separated on Bis-Tris gels and immunoblotted using the anti-HA antibody, or control antisera raised to TrxA (cytoplasmic protein), protein A (Spa, cell wall), or sortase A (SrtA, membrane). (*E*) Schematic representation of the toxicity experiments in C, and the inhibition of TspA_CT_ toxicity by the membrane-embedded immunity protein, TsaI. IM, inner membrane; OM, outer membrane; PP, periplasm.

Sequence analysis of TspA indicates that homologs are found across the *Staphylococci* (including *Staphylococcus argenteus*, *Staphylococcus epidermidis*, and *Staphylococcus lugdunensis*), in *Listeria* species and Enterococci, but does not provide clues about potential function. However, analysis of TspA using modeling programs predicts strong structural similarity to colicin Ia (Fig. 3*A*), a bacteriocidal protein produced by some strains of *Escherichia coli*. Colicin Ia has an amphipathic domain at its C terminus that inserts into the cytoplasmic membrane from the extracellular side to form a voltage-gated channel (30–32). Some limited structural similarity was also predicted with the type III secretion translocator protein YopB, which undergoes conformational changes to form pores in host cell membranes (33).

To investigate the function of TspA, DNA encoding full-length TspA or the C-terminal domain alone (TspA_CT_) was cloned into a tightly regulatable vector for expression in *E. coli*. Fig. 3*C* shows that production of TspA or TspA_CT_ did not affect *E. coli* survival. However, colicin Ia shows a sidedness for channel formation because it requires a transmembrane voltage for full insertion (34). We therefore targeted TspA_CT_ to the periplasm of *E. coli* by fusing to a Tat signal peptide (35, 36). Fig. 3*C* shows that this construct was toxic, and that toxicity was relieved when the Tat pathway was inactivated (Fig. 3*C*), consistent with the C-terminal domain of TspA exerting toxic activity from the periplasmic side of the membrane.

Bacterially produced toxins, particularly those that target other bacteria, are often coexpressed with immunity proteins that protect the producing cell from self-intoxication. For example, protection from colicin Ia toxicity is mediated by the membrane-bound Iia immunity protein (37). TspA is genetically linked to a repeat region of 10 genes encoding predicted polytopic membrane proteins with DUF443 domains (Fig. 3*B*). Topological analysis of these proteins predicts the presence of five transmembrane domains with an N_out_-C_in_ configuration. Consistent with this, Western blot analysis confirmed that a C-terminally HA-tagged variant of SAOUHSC_00585, which is encoded directly adjacent to TspA, localized to the membrane of *S. aureus* (Fig. 3*D*). To determine whether SAOUHSC_00585 offers protection against the toxicity of the TspA C-terminal domain, we coproduced the AmiAss–TspA_CT_ fusion alongside SAOUHSC_00585 in *E. coli*. Fig. 3*C* shows that coproduction of SAOUHSC_00585 offered protection of *E. coli*, particularly when it was constitutively expressed from the pSUPROM plasmid. SAOUHSC_00585 was subsequently renamed TsaI (TspA immunity protein) (Fig. 3*E*).

**Fig. 4.**
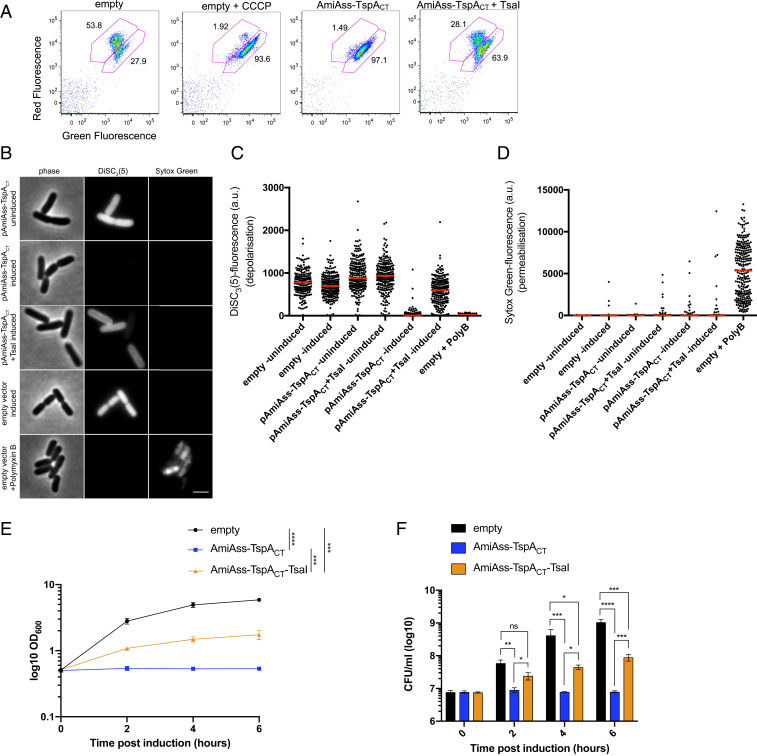
The C-terminal domain of TspA has bacteriostatic activity and disrupts the membrane potential. (*A*) *E. coli* MG1655 harboring pBAD18-Cm (empty), or pBAD18-Cm encoding AmiAss–TspA_CT_ or AmiAss–TspA_CT_/TsaI were grown in the presence of 0.2% l-arabinose for 1 h at which point they were diluted to 1 × 10^6^ cells per mL and supplemented with 30 μM DiOC_2_(3) for 30 min. One sample of MG1655 harboring pBAD18 (empty) was also supplemented with 5 μM CCCP at the same time as DiOC_2_(3) addition. Strains were analyzed by flow cytometry. (*B*–*D*) The same strain and plasmid combinations as *A* were grown in the presence (induced) or absence (uninduced) of 0.2% l-arabinose for 30 min, after which they were supplemented with DisC_3_(5) and Sytox green and (*B*) imaged by phase-contrast and fluorescence microscopy. (Scale bar: 3 μm.) (*C* and *D*) Fluorescence intensities of (*C*) DisC_3_(5) and (*D*) Sytox green for each sample was quantified using ImageJ. A control sample where Polymyxin B was added to the uninduced empty vector control for 5 min before supplemented with DisC_s_(5) and Sytox green was included in each experiment. (*E* and *F*) Growth of *E. coli* MG1655 harboring pBAD18-Cm (empty), or pBAD18-Cm encoding AmiAss–TspA_CT_ or AmiAss–TspA_CT_/TsaI upon induction with 0.2% l-arabinose. LB medium was inoculated with an overnight culture of *E. coli* strain MG1655 harboring the indicated constructs to a starting OD_600_ of 0.1. Cells were incubated at 37 °C and allowed to reach an OD_600_ of 0.5 (indicated by time 0) before supplementing the growth medium with 0.2% l-arabinose (inducing conditions). The growth was monitored every 2 h and the colony forming units at each time point was determined. Points and bars show mean ± SEM (*n* = 3 biological replicates). (*E*) Significance testing was performed by calculating the area under the curve (AUC) for each experimental replicate using GraphPad Prism 7.0 and then performing a one-way ANOVA with Sidak’s correction. (*F*) Significance testing performed using a one-way ANOVA with Sidak’s correction at each timepoint. *P < 0.05 **P < 0.01, ***P < 0.001, ****P < 0.0001, ns, not significant.

Pore-forming proteins are widely used as toxins to target either prokaryotic or eukaryotic cells (38, 39). To assess whether TspA has pore/channel-forming activity we investigated whether the production of AmiAss–TspA_CT_ in *E. coli* dissipated the membrane potential. Initially we used the BacLight assay, which is based on the dye 3,3′-diethyloxacarbocyanine iodide DiOC_2_(3) that exhibits green florescence in dilute solution but a red shift following membrane potential-driven accumulation in the bacterial cytosol. After sorting of *E. coli* by flow cytometry, the majority of cells harboring the empty vector exhibited red fluorescence, which shifted to green following treatment with the uncoupler carbonyl cyanide 3-chlorophenylhydrazone (CCCP). A similar shift in fluorescence was also observed when *E. coli* produced the AmiAss–TspA_CT_ fusion (Fig. 4*A*), indicative of loss of membrane potential. Coproduction of TsaI offered some protection from AmiAss–TspA_CT_-induced depolarization (Fig. 4*A*). We conclude that the C-terminal domain of TspA has membrane depolarizing activity.

Membrane depolarization may arise from the formation of ion-selective channels or larger, nonselective pores. To further investigate the mechanism of membrane depolarization, we used single-cell microscopy that combines the voltage-sensitive dye DisC_3_(5) with the membrane-impermeable nucleic acid stain Sytox green (40). *E. coli* cells incubated with Polymyxin B, which produces large ion-permeable pores in the *E. coli* cell envelope (41), showed strong labeling with Sytox green, indicative of permeabilization, coupled with very low DisC_3_(5) fluorescence (Fig. 4 *B*–*D*). In contrast, cells harboring the empty vector had high DisC_3_(5) fluorescence that was unaffected by supplementation with the inducer l-arabinose, and did not stain with Sytox green. Cells expressing the AmiAss–TspA_CT_ fusion following incubation with arabinose rapidly depolarized, as evidenced by the marked reduction in DisC_3_(5) fluorescence, but did not detectably stain with Sytox green, even after prolonged periods of incubation (Fig. 4 *B*–*D* and *SI Appendix*, Fig. S3). Therefore, it appears that TspA acts by triggering membrane depolarization but does so by forming ion channels rather than larger, nonselective pores in the *E. coli* inner membrane. Again, coproduction of TsaI significantly protected cells from AmiAss–TspA_CT_-induced depolarization, confirming that it acts as an immunity protein (Fig. 4 *B*–*D*).

Bacterial channel-forming toxins have been reported that have either bacteriocidal (42) or bacteriostatic (43) activity. To determine whether the C-terminal domain of TspA was bacteriocidal or bacteriostatic, the growth of *E. coli* producing AmiAss–TspA_CT_ was monitored. It was observed that upon production of AmiAss–TspA_CT,_ *E. coli* ceased to grow (Fig. 4*E*); however, quantification of the colony forming units (cfu) indicated that the cells did not lose viability (Fig. 4*F*), pointing to a bacteriostatic action of TspA. We conclude that the C-terminal domain of TspA is a channel-forming toxin with bacteriostatic activity that is neutralized by the action of TsaI.

### A Zebrafish Model for *S. aureus* Infection and T7SS Activity.

We next probed whether TspA was important for *S. aureus* virulence, initially through the development of an immunocompetent murine model of *S. aureus* pneumonia, which failed to reveal the impact of T7SS activity in vivo (*SI Appendix*, Fig. S4). Given that there are likely to be roles for the T7SS in bacterial competition as well as direct interaction with the host, we next developed a model where these two potentially confounding factors could be investigated. The zebrafish (*Danio rerio*), a widely used vertebrate model for development, has recently been adapted to study bacterial infection by human pathogens (44). The hindbrain ventricle offers a sterile compartment that can be used to follow bacterial interactions in vivo (45). We first assessed the utility of this infection model by testing the effect of dose and temperature on survival for *S. aureus* inoculated into the hindbrain ventricle of zebrafish larvae 3 d postfertilization (dpf) (*SI Appendix*, Fig. S5*A*). Clear dose-dependent zebrafish mortality was observed, with ∼90% of zebrafish surviving a low dose of *S. aureus* infection (7 × 10^3^ cfu), whereas only ∼55% survived a higher dose (2 × 10^4^ cfu) (*SI Appendix*, Fig. S5*B*). Although 28.5 °C is the optimum temperature for zebrafish larvae development, *S. aureus* has a temperature optimum of 30 to 37 °C for growth. In agreement with this, we observed significantly increased zebrafish mortality at 33 °C (relative to 28.5 °C) at high-dose infection (*SI Appendix*, Fig. S5*B*).

**Fig. 5.**
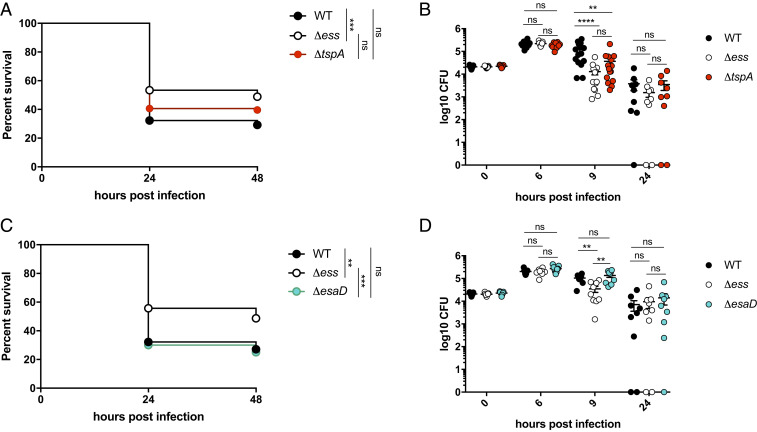
The T7SS contributes to virulence in a zebrafish infection model. (*A*) Survival curves of 3 dpf zebrafish *lyz*:dsRed larvae infected in the hindbrain ventricle with RN6390-gfp (WT) or otherwise isogenic Δ*ess*-gfp or Δ*tspA*-gfp strains at a dose of ∼2 × 10^4^ cfu and incubated at 33 °C for 48 hpi. Data are pooled from four independent experiments (*n* = 25 to 51 larvae per experiment). Results were plotted as a Kaplan–Meier survival curve and the *P* value between conditions was determined by log-rank Mantel–Cox test. (*B*) Enumeration of recovered bacteria at 0, 6, 9, or 24 hpi from zebrafish larvae infected with the same strains as *A*. Pooled data from three independent experiments. Circles represent individual larva, and only larvae that survived the infection were included. No significant differences observed between strains at 0, 6, or 24 hpi. Mean ± SEM also shown (horizontal bars). Significance testing was performed using a one-way ANOVA with Sidak’s correction at each timepoint. (*C*) Survival curves of 3 dpf zebrafish *lyz*:dsRed larvae infected in the hindbrain ventricle with RN6390-gfp (WT) or otherwise isogenic Δ*ess*-gfp, Δ*esaD*-gfp strains at a dose of ∼2 × 10^4^ cfu and incubated at 33 °C for 48 hpi. Data are pooled from three independent experiments (*n* = 26 to 32 larvae per experiment). Results are plotted as a Kaplan–Meier survival curve and the *P* value between conditions was determined by log-rank Mantel–Cox test. (*D*) Enumeration of recovered bacteria at 0, 6, 9, or 24 hpi from zebrafish larvae infected with the strains as *C*. Pooled data from three independent experiments. Circles represent individual larva, and only larvae having survived the infection were included. No significant differences observed between strains at 0, 6, or 24 hpi. Mean ± SEM also shown (horizontal bars). Significance testing was performed using a one-way ANOVA with Sidak’s correction at each timepoint. **P < 0.01, ***P < 0.001, ****P < 0.0001, ns, not significant.

We next assessed whether there was a role for the T7SS in zebrafish mortality. For these experiments, larvae at 3 dpf were inoculated in the hindbrain ventricle with 2 × 10^4^ cfu of RN6390 or an isogenic strain, RN6390 Δ*ess*, lacking all 12 genes (*esxA* through *esaG*) at the *ess* locus (3), and incubated at 33 °C. We routinely observed that zebrafish mortality was significantly reduced, at both 24 and 48 h postinoculation (hpi), for zebrafish infected with the RN6390 Δ*ess* strain compared to the wild-type (Fig. 5 *A* and *C* and *SI Appendix*, Fig. S5*C*). In agreement, total bacterial counts of infected zebrafish revealed that following an initial period of 6 h, where both strains replicated in a similar manner, there was a significant decrease in recovery of the Δ*ess* strain compared to the wild-type after 9 h (Fig. 5 *B* and *D* and *SI Appendix*, Fig. S5*D*), suggesting that bacteria lacking the T7SS are more rapidly cleared in vivo. We also tested a second *S. aureus* strain, COL, in this assay. COL was only weakly virulent at 24 hpi, but at high dose substantial mortality was seen after 48 h (*SI Appendix*, Fig. S6*A*). As before, zebrafish mortality was at least in part dependent on a functional T7SS (*SI Appendix*, Fig. S6*B*), although we observed no difference in bacterial burden between the wild-type and Δ*essC* strain at the timepoints sampled (*SI Appendix*, Fig. S6*C*). We conclude that the T7SS plays a role in virulence of *S. aureus* in this zebrafish infection model.

In addition to TspA, a second T7SS-secreted toxin, EsaD [also called EssD (9, 46)], a nuclease, has been identified in some *S. aureus* strains. EsaD was shown to inhibit growth of a competitor *S. aureus* strain in vitro (6), but has also been directly implicated in virulence through modulation of cytokine responses and abscess formation (9, 46). We therefore determined whether TspA or EsaD was required for virulence in the zebrafish infection model. Infection of larvae with strain RN6390 lacking TspA resulted in levels of mortality intermediate between the wild-type and Δ*ess s*train (Fig. 5*A*), and a significantly reduced bacterial burden relative to the wild-type strain at 9 hpi (Fig. 5*B*). In contrast, no difference was observed in either zebrafish mortality (Fig. 5*C*) or bacterial burden (Fig. 5*D*) between infection with RN6390 and an isogenic *esaD* mutant, indicating no detectable role of EsaD in virulence. Taking these data together, we conclude that zebrafish infection can be used to investigate the role of T7SS effectors in vivo, and that TspA (but not EsaD) contributes to T7SS-mediated bacterial replication in vivo.

Previous studies have shown that the T7SS of *S. aureus* is involved in modulating the murine host immune response (9, 46). To test whether altered immune responses mediate the increased clearance of the Δ*ess* and Δ*tspA* deletion strains at 9 hpi, we investigated the role of the T7SS in the zebrafish larval cytokine response during *S. aureus* infection in vivo (*SI Appendix*, Fig. S7). The expression of two host proinflammatory markers IL-8 (*cxcl8)* and IL-1β (*il-1b*) were quantified using qRT-PCR in larvae infected with 2 × 10^4^ cfu of RN6390 wild-type, Δ*ess*, Δ*tspA*, and Δ*esaD* strains. In comparison to PBS-injected larvae, *S. aureus* infection caused a robust increase in both *cxcl8* and *il-1b* expression at 6 hpi (when the bacterial burden among strains was similar) (*SI Appendix*, Fig. S7). However, no significant difference in gene expression was observed among larvae infected with wild-type and any of the three deletion strains (Δ*ess*, Δ*tspA*, and Δ*esaD*) (*SI Appendix*, Fig. S7).

Neutrophils represent the first line of defense against *S. aureus* infection (47) and the recently discovered substrate of EssC variant 2 strains, named EsxX, has been implicated in neutrophil lysis, therefore contributing to evasion of the host immune system (48). In contrast, the T7SS of *Mycobacterium tuberculosis* (ESX-1) is associated with manipulation of the inflammatory response during infection, allowing for bacterial replication in macrophages (49–52). To investigate whether the *S. aureus* T7SS modulates interaction with leukocytes, we analyzed the recruitment of immune cells to the hindbrain using two transgenic lines in which dsRed is expressed specifically in neutrophils [Tg(*lyz*::dsRed)] or mCherry is expressed specifically in macrophages [Tg(*mpeg*::Gal4-FF)^gl25^/Tg(*UAS*-*E1b*::*nfsB*.mCherry)^c264^, herein Tg(*mpeg1*::G/U::mCherry)]. Zebrafish larvae were infected with RN6390 wild-type, Δ*ess*, and Δ*tspA* strains in the hindbrain ventricle at 3 dpf and imaged under a fluorescent stereomicroscope at 0, 3, and 6 hpi in order to monitor neutrophil (*SI Appendix*, Fig. S8 *A* and *B*) and macrophage (*SI Appendix*, Fig. S8 *C* and *D*) behavior. In zebrafish larvae infected with *S. aureus*, a significant increase in neutrophil recruitment to the hindbrain ventricle was detected in comparison to PBS-injected larvae at both 3 and 6 hpi (*SI Appendix*, Fig. S8*B*). However, no difference in neutrophil recruitment to the Δ*ess* and Δ*tspA* strains relative to wild-type was detected at any of the time points tested (*SI Appendix*, Fig. S8*B*). Similar to the neutrophil recruitment experiments, a significant increase in macrophage recruitment to the site of *S. aureus* infection was observed when compared to PBS-injected larvae at 3 hpi (*SI Appendix*, Fig. S8*D*). However, there was no significant difference in macrophage recruitment among the wild-type and T7SS mutant strains (*SI Appendix*, Fig. S8*D*).

### T7SS-Dependent Bacterial Competition In Vivo.

Although TspA is required for optimal *S. aureus* virulence in the zebrafish model, the observed toxicity when heterologously produced in *E. coli* coupled with the presence of immunity genes encoded downstream of *tspA* strongly suggested that secreted TspA may also have antibacterial activity. Previously, to observe antibacterial activity of the nuclease EsaD in laboratory growth media required the toxin to be overproduced from a multicopy plasmid (6). However, zebrafish larvae have recently been adapted to study bacterial predator–prey interactions (45), and we reasoned that since the T7SS was active in our zebrafish infection model it may also provide a suitable experimental system to investigate the impact of T7-mediated bacterial competition in vivo.

**Fig. 6.**
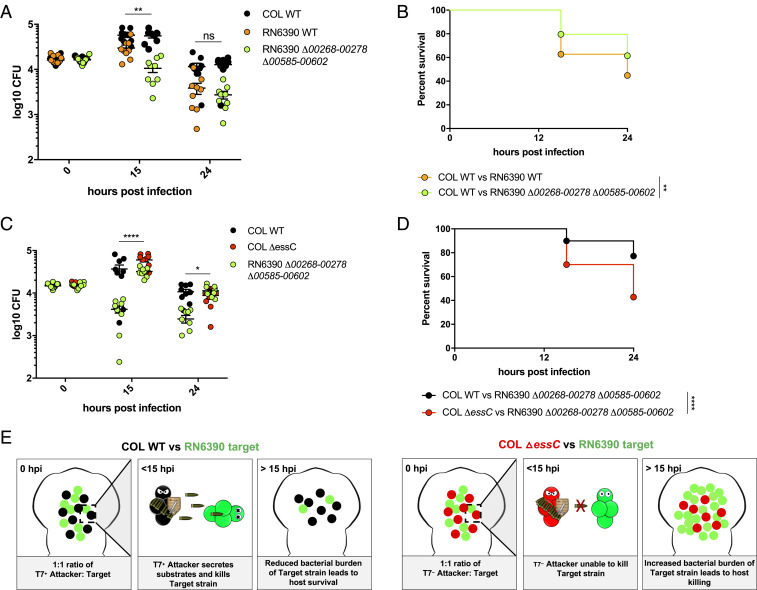
Development of an in vivo model to study bacterial competition. Wild-type AB zebrafish larvae at 3 dpf were coinfected with a 1:1 mix of an attacker strain (either COL-mCherry [WT] or COL Δ*essC*-mCherry as indicated) and a target strain (either RN6390-gfp (WT) or RN6390 Δ*00268-278* Δ*00585-00602*-gfp, as indicated). (*A* and *C*) Enumeration of recovered attacker and prey bacteria from zebrafish larvae at 0, 15, or 24 hpi. Pooled data from three independent experiments. Mean ± SEM also shown (horizontal bars). Significance testing performed by unpaired *t* test. (*B* and *D*) Survival curves of zebrafish injected with the indicated strain pairs. Data are pooled from three independent experiments. Results are plotted as a Kaplan–Meier survival curve and the *P* value between conditions was determined by log-rank Mantel–Cox test. *P < 0.05, **P < 0.01, ****P < 0.0001, ns, not significant. (*E*) Model highlighting the role for the T7SS in competition in vivo.

In these experiments we used COL as the attacker strain and RN6360 and its derivatives as the target; it should be noted that these strains produce the same TspA and EsaD isoforms, as well as similar suites of immunity proteins. COL was coinoculated into the hindbrain ventricle, at a 1:1 ratio, with either RN6390 or an isogenic strain lacking all potential immunity proteins for EsaD and TspA (FRU1; RN6390 Δ*saouhsc00268-00278*, Δ*saouhsc00585-00602*). A significant reduction in recovery of the target strain lacking immunity genes was observed compared to the isogenic parental strain at 15 h postinfection (Fig. 6*A*). Conversely, there was significantly greater zebrafish mortality at 24 h after coinoculation of COL with the wild-type RN6390 than the immunity mutant strain (Fig. 6*B*). Since COL is almost completely avirulent at this time point (*SI Appendix*, Fig. S6), we infer that mortality arises from RN6390, and as the wild-type strain survives better than the immunity deletion strain when coinoculated with COL, this accounts for the greater zebrafish mortality.

To confirm that reduced growth of the RN6390 immunity mutant strain was dependent upon a functional T7SS in the attacking strain, we repeated the coinoculation experiments using a T7 mutant strain of COL (COL Δ*essC*). The RN6390 immunity mutant strain showed significantly higher recovery after 15 h in the presence of the COL T7 mutant strain than wild-type COL (Fig. 6*C*) and accordingly this was linked with reduced zebrafish survival (Fig. 6*D*). Collectively, these data highlight the utility of zebrafish for investigating *S. aureus* competition in vivo, and demonstrate that bacterial competition and zebrafish mortality is dependent on a functional T7SS in the attacking strain (COL). This is outlined in the schematic shown in Fig. 6*E*. Conversely, the ability of the prey strain (RN6390) to survive T7-dependent killing is dependent upon the immunity proteins against EsaD and TspA, because when these are not present, fewer bacteria are recovered.

**Fig. 7.**
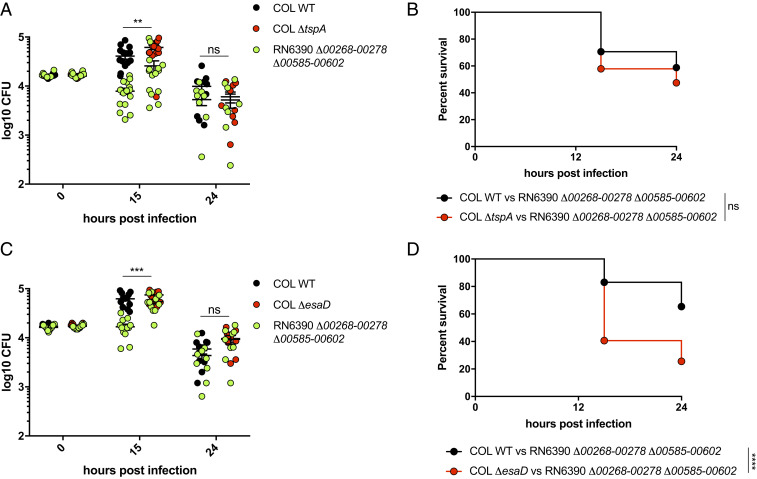
TspA and EsaD dependent bacterial competition in vivo. Wild-type AB zebrafish larvae at 3 dpf were coinfected with a 1:1 mix of an attacker strain (either COL-mCherry [WT], COL Δ*tspA*-mCherry or COL Δ*esaD*-mCherry as indicated) and a target strain (RN6390 Δ*00268-278* Δ*00585-00602*-gfp). (*A* and *C*) Enumeration of recovered attacker and prey bacteria from zebrafish larvae at 0, 15, or 24 hpi. Pooled data from three independent experiments. Mean ± SEM also shown (horizontal bars). Significance testing performed by unpaired *t* test. (*B* and *D*) Survival curves of zebrafish injected with the indicated strain pairs. Data are pooled from three independent experiments. Results are plotted as a Kaplan–Meier survival curve and the *P* value between conditions was determined by log-rank Mantel–Cox test. **P < 0.01, ***P < 0.001, ****P < 0.0001, ns, not significant.

Finally, we investigated which of the EsaD and TspA toxins was responsible for interstrain competition by using variants of COL deleted for either *tspA* or *esaD* as the attacking strain. It was seen that in the absence of either TspA (Fig. 7*A*) or EsaD (Fig. 7*C*), there was a significant increase in recovery of the RN6390 Δ*saouhsc00268-00278*, Δ*saouhsc00585-00602* prey strain, indicating that each of these toxins has activity against the target strain. However, there was a more pronounced increase in zebrafish mortality when the attacker strain lacked *esaD* than *tspA* (compare Fig. 7 *B* and *D*), suggesting that EsaD has the more potent antibacterial activity in these conditions.

## Discussion

Here we have taken an unbiased approach to discover substrates of the T7SS in *S. aureus* RN6390, identifying the LXG-domain protein, TspA. TspA localizes to the cell envelope and has a toxic C-terminal domain that has membrane-depolarizing activity. While all other previously identified T7 substrates are encoded at the *ess* locus and are associated with specific *essC* subtypes (21, 48), TspA is encoded elsewhere on the genome, and is conserved across all *S. aureus* strains. This suggests TspA plays a key role in *S. aureus*, and indeed we show using a zebrafish infection model that it contributes to T7SS-mediated bacterial replication in vivo.

Pore- and channel-forming toxins are key virulence factors produced by many pathogenic bacteria (53) that can act both extracellularly to form pores in eukaryotic cells, like some bacterial hemolysins (54), or intracellularly for example by altering permeability of the phagosome, like the pore-forming toxin Listeriolysin-O, or the type III secretion system effector VopQ (55, 56). The *S. aureus* T7SS has been strongly linked with modulating the host innate immune response (9, 46). However, we did not observe any significant difference between wild-type and T7SS mutant strains in modulating cytokine expression and phagocyte recruitment in zebrafish larvae. Although the precise mechanism by which the T7SS and TspA interacts with host cells remains to be determined, we hypothesize that the T7SS plays a role after phagocytosis by immune cells to influence intracellular survival. Future work using high-resolution single-cell microscopy would allow for individual *S. aureus* cells, as well as their interactions with neutrophils and macrophages, to be tracked in vivo.

Sequence alignments indicate that the C-terminal domain of TspA is polymorphic across *S. aureus* strains (*SI Appendix*, Fig. S9) and structural modeling of TspA suggests homology to colicin Ia. Colicin Ia is a toxin that forms voltage-gated ion channels in the plasma membrane of sensitive *E. coli* strains. The formation of these channels results in lysis of target bacteria (31, 42). Heterologous expression of the C-terminal predicted channel-forming domain of TspA was shown to dissipate the membrane potential of *E. coli* when it was targeted to the periplasm, probably through formation of an ion channel. Unlike colicin Ia, however, heterologous production of the TspA toxin domain was associated with a bacteriostatic rather than a bacteriocidal activity. Colicins and pyocins are also examples of polymorphic toxins (39) and the producing cells are generally protected from colicin-mediated killing by the presence of immunity proteins (37). A cluster of membrane proteins from the DUF443 domain family are encoded downstream of *tspA*, and we show that at least one of these (SAOUHSC_00585; TsaI) acts as an immunity protein to TspA by protecting *E. coli* from TspA-induced membrane potential depletion.

Polymorphic toxins are frequently deployed to attack competitor bacteria in polymicrobial communities (38), and there is growing evidence that a key role of the T7SS in some bacteria is to mediate inter- and intraspecies competition (6, 7). In addition to TspA, many commonly studied strains of *S. aureus*, including RN6390 and COL, also secrete a nuclease toxin, EsaD (6). We adapted our zebrafish larval infection model to assess the role of the T7SS and the secreted toxins TspA and EsaD in intraspecies competition. We observed that strain COL was able to outcompete RN6390 in a T7SS-dependent manner in these experiments, provided that RN6390 was lacking immunity proteins to TspA and EsaD. Experiments with individual COL attacker strains deleted for either *tspA* or *esaD* showed that each of the toxins contributed to the competitiveness of COL in these assays. As *S. aureus* is a natural colonizer of human nares and can also exist in polymicrobial communities in the lungs of cystic fibrosis patients, we suggest that secreted T7 toxins including TspA allow *S. aureus* to establish its niche by outcompeting other bacteria. Indeed, the observation that the T7SS gene cluster is highly up-regulated in the airways of a cystic fibrosis patient (5) would be consistent with this hypothesis.

LXG domain proteins appear to form a large substrate family of the firmicutes T7SS. Three LXG domain proteins of *S. intermedius* have been shown to mediate contact-dependent inhibition (7), and the association of TspA with the *S. aureus* cell envelope would also imply that toxicity is contact-dependent. The LXG domain is predicted to form an extended helical hairpin, which could potentially span the cell wall, displaying the toxin domain close to the surface. How any of these toxin domains reach their targets in the prey cell is not clear. One possibility is that the toxin domain is taken up into the target cell upon interaction with a surface receptor, as observed for the type V-dependent contact inhibition systems in gram-negative bacteria (57, 58). During this process the CdiA protein, which also has a C-terminal toxin domain, is proteolyzed, releasing the toxin to interact with its cellular target (59). Further work would be required to decipher the mechanisms by which LXG toxins access target cells and whether the toxin domains undergo proteolysis to facilitate cellular entry.

In conclusion, channel-forming toxin substrates have been associated with other protein secretion systems (43, 55–57), but this is unique in being functionally described for the T7SS. To our knowledge it is only the second bacterial exotoxin identified to have a phenotype in both bacterial competition and virulence assays, after VasX from *Vibrio cholerae* (60, 61).

## Materials and Methods

### Bacterial Strains, Plasmids, and Growth Conditions.

Construction of strains and plasmids, and growth conditions are described in *SI Appendix*, *SI Materials and Methods*. Plasmids and strains used in this study are given in *SI Appendix*, Tables S1 and S2.

### Mass Spectrometry Data Analysis and Label-Free Quantitation.

Preparation of *S. aureus* culture supernatants for proteomic analysis is detailed in *SI Appendix*, *SI Materials and Methods*. Sample preparation and mass spectrometry analysis was performed similar to previously described work (62–65) and detailed methods are described in *SI Appendix*, *SI Materials and Methods*.

### Cell Fractionation and Western Blotting.

Preparation of *S. aureus* cell and supernatant samples for Western blotting, and subcellular fractionation of *S. aureus* into the cell wall, membrane, and cytoplasmic fractions were as described previously (3). Preparation of urea-washed membrane fractions was adapted from Keller et al. (66). Briefly, broken cell suspensions were thoroughly mixed with a final concentration of 4 M urea and incubated for 20 min at room temperature. Membranes were harvested by ultracentrifugation (227,000 × *g*, 30 min). The supernatant was retained as the urea-treated cytoplasmic fraction and the membrane pellet resuspended in 1× PBS, 0.5% Triton X-100. For spheroplast preparation, the method of Götz et al. (67) was adapted. Briefly, strains were cultured as described above, cells were harvested at OD_600_ of 2.0, and resuspended in Buffer A (0.7 M sucrose, 20 mM maleate, 20 mM MgCl_2_, pH 6.5). Lysostaphin and lysozyme were added at 20 μg/mL and 2 mg/mL final concentration, respectively, and cells incubated at 37 °C for 1 h. Cell debris was pelleted by centrifugation (2,500 × *g* for 8 min) and the resulting supernatant centrifuged at 16,000 × *g* for 10 min to pellet the spheroplasts. Spheroplasts were resuspended in Buffer A and treated with increasing concentrations of Proteinase K on ice for 30 min. Next, 0.5 mM phenylmethylsulfonyl fluoride was added to terminate the reaction and samples mixed with 4× Nu PAGE LDS sample buffer and boiled for 10 min prior to further analysis. Western blotting was performed according to standard protocols using the following antibody dilutions α-EsxA (3) 1:2,500; α-EsxC (3) 1:2,000; α-EssB (3) 1:10,000; α-TrxA (68) 1:25000; α-SrtA (Abcam, catalog number ab13959) 1:3,000; α-HA (HRP-conjugate, Sigma catalog number H6533) α-Myc (HRP-conjugate, Invitrogen catalog number R951-25) 1:5,000; and goat anti Rabbit IgG HRP conjugate (Bio-Rad, catalog number 170-6515) 1:10,000.

### Bacterial Membrane Potential Detection.

To assess bacterial membrane potential, the method of Miyata et al. (69) was adapted, using the BacLight bacterial membrane potential kit (Invitrogen). Detailed methods to assess both bacterial membrane potential and permeabilization are described in *SI Appendix*, *SI Materials and Methods*.

### Zebrafish Infection.

Wild-type (AB strain) or transgenic Tg(*lyz*::dsRed)^*nz50*^ (70) zebrafish were used for all survival experiments. Embryos were obtained from naturally spawning zebrafish, and maintained at 28.5 °C until 3 dpf in embryo medium (0.5× E2 medium supplemented with 0.3 g/mL methylene blue) (71). For injections, larvae were anesthetized with 200 µg/mL tricaine (Sigma-Aldrich) in embryo medium. Hindbrain ventricle infections were carried out at 3 dpf and incubated at 33 °C unless specified otherwise. Bacteria were subcultured following overnight growth until they reached OD_600_ of 0.6. For injection of larvae, bacteria were recovered by centrifugation, washed, and resuspended in 1× PBS, 0.1% phenol red, 1% polyvinylpyrrolidone to the required cfu/mL. Anesthetized larvae were microinjected in the hindbrain ventricle with 1 to 2 nL of bacterial suspension. At the indicated times, larvae were killed in tricaine, lysed with 200 μL of 0.4% Triton X-100, and homogenized mechanically. Larval homogenates were serially diluted and plated onto TSB agar. Only larvae having survived the infection were included for enumeration of cfu. For zebrafish virulence assays, all *S. aureus* strains were chromosomally tagged with GFP, which included RN6390 wild-type, and isogenic Δ*ess*, Δ*tspA*, and Δ*esaD* strains. For in vivo competition experiments, COL (attacker) strains were chromosomally tagged with mCherry and RN6390 (target) strains with GFP. Attacker and target strains were subcultured, harvested, and resuspended in PBS as above. Attacker and target strains were mixed at a 1:1 ratio and injected in the hindbrain ventricle, with 1 to 2 nL of bacterial suspension. Larvae were killed at 15 hpi or 24 hpi, serially diluted and plated on TSB agar, and attacker and target strains were enumerated by fluorescence (GFP and mCherry). Quantitative reverse transcription PCR and *S. aureus*-leukocyte microscopy methods are described in *SI Appendix*, *SI Materials and Methods*. Animal experiments were performed according to the Animals (Scientific Procedures) Act 1986 and approved by the UK Home Office (Project licenses: PPL P84A89400 and P4E664E3C).

## Supplementary Material

Supplementary File

Supplementary File

## Data Availability

Raw mass spectrometry data that support the findings of this study have been deposited to the ProteomeXchange Consortium via the PRIDE (72) partner repository (dataset identifier PXD011358). All other data supporting the findings of this study are available within the paper and *SI Appendix*.
