# Supplementary material for: A membrane-depolarizing toxin substrate of the Staphylococcus aureus type VII secretion system mediates intraspecies competition
Source: Proc Natl Acad Sci U S A. 2020 Aug 7;117(34):20836–47. doi: 10.1073/pnas.2006110117 (PMC7456083; doi:10.1073/pnas.2006110117)
Supplement: Supplementary File [file pnas.2006110117.sapp.pdf]

PNAS
www.pnas.org

Supplementary Information for

A membrane-depolarizing toxin substrate of the *Staphylococcus aureus* Type VII secretion system mediates intra-species competition

Fatima R. Ulhuq, Margarida C. Gomes, Gina Duggan, Manman Guo, Chriselle Mendonca, Grant Buchanan, James D. Chalmers, Zhenping Cao, Holger Kneuper, Sarah Murdoch, Sarah Thomson, Henrik Strahl, Matthias Trost, Serge Mostowy and Tracy Palmer

Corresponding authors:
Tracy Palmer and Serge Mostowy

Email: tracy.palmer@newcastle.ac.uk or serge.mostowy@lshtm.ac.uk

This PDF file includes:

- Tables S1 to S3
- SI Materials and Methods
- Figures S1 to S9
- SI References

Other supplementary materials for this manuscript include the following:

- Dataset S1

www.pnas.org/cgi/doi/10.1073/pnas.2006110117

SI Tables

Table S1. Strains used in this study

Strain	Relevant genotype or description	Source or reference
S. aureus strains		
RN6390	NCTC8325 derivative, *rbsU*, *tcaR*, cured of φ11, φ12, φ13	Reference[1]
RNΔ*essC*	As RN6390, Δ*essC*	Reference[2]
RNΔ*ess*	Complete deletion from *esxA* – *esaG*	Reference[2]
RNΔ*esaD*	As RN6390, Δ*esaD*	Reference[3]
RNΔ*tspA*	As RN6390, Δ*tspA* (*saouhsc00584)*	This work
RNΔ*saouhsc00268-00278*	As RN6390, Δ*esaD-saouhsc00278*	Reference[3]
RNΔ*saouhsc00585-00602*	As RN6390, Δ*saouhsc00585-saouhsc00602*	This work
FRU1	RN6390 Δ*saouhsc00268-00278*, Δ*saouhsc00585-00602*	This work
RN6390*::ermC*	As RN6390, with *ermC* resistance gene chromosomal insertion	Reference[3]
RNΔ*saouhsc00268-00278::ermC*	As RN6390, Δ*esaD-saouhsc00278* with *ermC* resistance gene from RN6390*::ermC* (phage ϕ11 transduction)	This work
FRU1*::ermC*	RN6390 Δ*saouhsc00268-00278*, Δ*saouhsc00585-00602* with *ermC* resistance gene from RN6390*::ermC* (phage ϕ11 transduction)	This work
RN6390::GFP	As RN6390, with markerless GFP insertion	This work
RNΔ*saouhsc00268-00278::gfp*	As RNΔ*esaD-saouhsc00278,* with markerless GFP insertion	RNΔ*saouhsc00268-00278*
RNΔ*saouhsc00585-00602::gfp*	As RNΔ*saouhsc00585-00602*, with markerless GFP insertion	This work

FRU1::*gfp*	RN6390 Δ*saouhsc00268-00278*, Δ*saouhsc00585-00602*, with markerless GFP insertion	This work
COL	MRSA, *agr*	Reference[4]
COLΔ*essC*	As COL, Δ*essC*	This work
COLΔ*esaD*	As COL, Δ*esaD*	This work
COLΔ*tspA*	As COL, Δ*tspA* (*sacol0643*)	This work
COL::*mCherry*	As COL, with markerless mCherry insertion	This work
COLΔ*essC::mCherry*	As COL, Δ*essC* with markerless mCherry insertion	This work
COLΔ*esaD::mCherry*	As COL, Δ*esaD* with markerless mCherry insertion	This work
COLΔ*tspA::mCherry*	As COL, Δ*tspA* (*sacol0643*) with markerless mCherry insertion	This work
E. coli strains		
JM110	*rpsL thr leu thi lacY galK galT ara tonA tsx dam dcm glnV44 Δ(lac-proAB)* e14- [F' *traD36 proAB*$^+$ *lacI*^{q} *lacZ*ΔM15] *hsdR17*(rK$^-$mK$^+$)	Stratagene
MG1655	*E. coli K-12, F−, λ−, ilvG, rfb-50, rph-1*	Reference[5]
SG3000	As MG1655, Δ*tatABCD*	Reference[6]

Table S2. Plasmids used in this study

Plasmid	Relevant genotype or description	Source or reference
pIMAY	*E. coli/S. aureus* shuttle vector, temperature sensitive, cml^r	Reference[7]
pIMAY-esaD	pIMAY carrying *esaD* deletion allele	Reference[3]
pIMAY-essC	pIMAY carrying *essC* deletion allele	Reference[2]
pIMAY-saouhsc00268-00278	pIMAY carrying *esaD-saouhsc00278* deletion allele	Reference[3]
pIMAY-tspA	pIMAY carrying *tspA* (*saouhsc00584*) deletion allele	This work
pIMAY-saouhsc00585-00602	pIMAY carrying *saouhsc00585-saouhsc00602* deletion allele	This work
pTH100	Plasmid for markerless integration of GFP into *S. aureus*	Reference[8]
pRN111	Plasmid for markerless integration of mCherry into *S. aureus*	Reference[8]
pBAD18-cm	Glucose-repressible/arabinose inducible vector; cml^r	Reference[9]
pBAD18-TspA	pBAD18-Cm producing TspA	This work
pBAD18-$TspA_{CT}$	pBAD18-Cm producing amino acids 218-469 of TspA	This work
pBAD18-AmiAss-$TspA_{CT}$	pBAD18-Cm producing *E. coli* AmiA signal sequence fused to amino acids 218-469 of TspA.	This work
pBAD18-AmiAss-$TspA_{CT}$ + TsaI	As pBAD18-AmiAss-$TspA_{CT}$ but also producing TsaI	This work
pSU-PROM	Cloning vector for expression of genes under the control of the *tat* promoter; Km^R	Reference[10]
pSU-PROM-TsaI	pSU-PROM producing TsaI	This work
pRAB11	*E. coli/S. aureus* shuttle vector, inducible protein expression, amp^r, cml^r	Reference[11]
pRAB11-TspA-Myc	pRAB11 producing C-terminally Myc-tagged TspA	This work
pRAB11-02448-HA	pRAB11 producing C-terminally HA-tagged SAOUHSC_02448	This work
pRAB11-00406-Myc	pRAB11 producing C-terminally Myc-tagged SAOUHSC_00406	This work
pRAB11-00389-HA	pRAB11 producing C-terminally HA-tagged SAOUHSC_00389	This work
pRAB11-00585-HA	pRAB11 producing C-terminal HA-tagged SAOUHSC_00585	This work
pRMC2	*E. coli-S. aureus* shuttle vector, inducible protein expression, amp^r, cml^r	Reference[12]
pRMC2 -SsaA-HA	pRMC2 producing C-terminally HA-tagged SsaA	This work

Table S3. Oligonucleotides and cloning strategies used in this study

Primer	Nucleotide Sequence (5'-3')
TspA A1	TAGGTACCGCTAATACATGCACGGC
TspA A2	CGCCCATTTCATCGGCATGCTCCTTTTC
TspA B1	ATGCCGATGAAATGGGCGTGGTGAGTT
TspA B2	CCGGTACCTGCTTTTTAAGTTTGGCATA
TspA out1	TCGCAAAGCAATATCCAC
TspA out2	TTGGTACCAATGGGGCATTTACGA
tspA cmyc fw	GCGCGGTACCAGGAGGTTTCTAGTTATGAGTATTGACATGTATTTAG AC
tspA cmyc rv	GCGCGAGCTCTCACAGATCCTCTTCTGAGATGAGTTTTTGTTCCCAC GCCCATTTCATTGGATTTATATG
02448 cha fw	GCGCGGTACCAGGAGGTTTCTAGTTATGGGAGTTAAAAGTGTG
02448 cha rv	GCGCGAGCTCTTATGCATAATCTGGAACATCATATGGATATTTTTTC C ATAAGAAGTC
00406 cmyc fw	GCGCGGTACCAGGAGGTTTCTAGTTATGTTGAGTAGGAAG
00406 cmyc rv	GCGCGAGCTCTTACAGATCCTCTTCTGAGATGAGTTTTTGTTCTAGT A ATCCACCTATTTGTG
00389 cha fw	GCGCGGTACCAGGAGGTTTCTAGTTATGAAAATAACAACGATTGC
00389 cha rv	GCGCGAGCTCTTATGCATAATCTGGAACATCATATGGATATTTTATAT TCACTTCAATG
00585 cha fw	GCGC AGATCT AGGAGG TTT CTA GTT ATG TTT TTA ATA TTA AGG TT
00585 cha rv	GCGCGAATTCTTAAGCATAATCTGGAACATCATATGGATATTGCTTTT TAAGTTTGGCATAAAC
pRMC2-ssaA-bglII-for	GGAGATCTAGAGTGTTTTGATTATTGGGA
pRMC2- SSaA-HA-rev-EcoRI	GGGAATTCTTATGCATAATCTGGAACATCATATGGATAATGAATGAA ATTATATGAACC
AmiAss fw	GCGCGCTAGCCAGAGGAGGAGCCATGAGCACTTTTAAACCAC
AmiAss rv	GCGCGGTACCGTCTTTGGCGATGGCTTGCGAC
tspA fl fw	GCGCTCTAGACAGAGGAGGAGCCATGAGTATTGACATGTATTTAGA C
tspA fl rv	GCGCGTCGACTTACCACGCCCATTTCATTGG
tspA cp fw	GCGCTCTAGACAGAGGAGGAGCCATGATTGAACATAAAGCAGAGAA AG
tspA pp fw	GCGCTCTAGAATTGAACATAAAGCAGAGAAAG
tsaI fw	GCGCGTCGACCAGAGGAGGAGCCATGCTTTTTAATATTAAGG
tsaI rv	GCGCGCATGCTTATTGCTTTTTAAGTTTGGCATAAAC
pSU-tsaI fw	GCGC GGATCC ATG CTT TTT AAT ATT AAG G
pSU-tsaI rv	GCGC CTCGAG TTATTG CTT TTT AAG TTT GGC

saouhsc00585-00602 A1	GCGCGAATTCCAGGTGGAGTGAAAGGCCCAGC
saouhsc00585-00602 A2	CTATTGGTTTTTATTAAAAAGCAAAACTCACCAC
saouhsc00585-00602 B1	TTGCTTTTTAATAAAAACCAATAGAAATTACCAA
saouhsc00585-00602 B2	GCGCGAGCTCCCGGTTGATTGTTCTGATGTAC
saouhsc00585-00602 out1	GCATATGCCAAAGAACATCCAG
saouhsc00585-00602 out2	GCTCTTGTAATGCTGCAACTGC
eef1a1a for	AAGCTTGAAGACAACCCCAAGAGC
eef1a1a rev	ACTCCTTTAATCACTCCCACCGCA
cxcl8 for	TGTGTTATTGTTTTCCTGGCATTTC
cxcl8 rev	GCGACAGCGTGGATCTACAG
il1b for	GAACAGAATGAAGCACATCAAACC
il1b rev	ACGGCACTGAATCCACCAC

SI Materials and Methods

Bacterial strains, plasmids and growth conditions. All strains and plasmids used in this study are given in *SI Appendix*, Tables S1 and S2. *S. aureus* strain RN6390[1] and its Δ*essC*[2], Δ*esaD*[3], Δ*SAOUHSC_00268-00278*[3] and Δ*ess* (Δ*esxA*-*esaG*)[2] derivatives along with strain COL[4] have been described previously. An in-frame deletion of *tspA* (*SAOUHSC_00584*) in RN6390 was constructed by allelic exchange using plasmid pIMAY (*SI Appendix*, Table S2)[4]. The upstream and downstream regions including the start codon and last six codons were amplified from RN6390 genomic DNA using primers listed in *SI Appendix*, Table S3 and were cloned into pIMAY and introduced onto the chromosome by recombination as described previously[4]. For deletion of *tsaI* and its homologues, the upstream regions of *SAOUHSC_00585* including its first four codons and the downstream regions of *SAOUHSC_00602* including its last four codons were amplified from RN6390 genomic DNA, cloned into pIMAY and was introduced into strain RN6390 Δ*SAOUHSC_00268-00278* to generate strain FRU1 (as RN6390 Δ*SAOUHSC_00268-00278*, Δ*SAOUHSC_00585-00602*). For in frame deletion of *esaD* (SACOL0281) and *tspA* (SACOL0643) in strain COL, constructs pIMAY-esaD[3] and pIMAY-tspA were used, following the protocol of Monk *et al.*[7]. Derivatives of strains harboring markerless *gfp* or *mCherry* chromosomal insertions were constructed according to de Jong *et al.*[8] using plasmids pTH100 and pRN111, respectively. *E. coli* strain JM110 was used for cloning purposes and MG1655[5] and its isogenic Δ*tatABCD* derivative SG3000[6] was used for toxicity assays.

All oligonucleotides used in this study are listed in *SI Appendix*, Table S3, and RN6390 chromosomal DNA was used as template unless otherwise stated. Plasmid pRAB11-tspA-myc encodes TspA with a C-terminal Myc tag in pRAB11[11] and was constructed following amplification with primers tspA cmyc fw and tspA cmyc rv. Plasmid pRAB11-

02448-ha produces SAOUHSC_02448 with a C-terminal HA tag from vector pRAB11 and the encoding gene was amplified using primers 02448 cha fw and 02448 cha rv. Plasmid pRAB11-00389-ha codes for SAOUHSC_00389 with a C-terminal HA tag in pRAB11 and was constructed following amplification with primers 00389 cha fw and 00389 cha rv. Plasmid pRAB11-00406-myc encodes SAOUHSC_00406 with a C-terminal Myc tag in pRAB11 and was constructed following amplification with primers 00406 cmyc fw and 00406 cmyc rv. In each case the amplified gene is preceded by the *esxA* RBS (AGGAGGTTTCTAGTT), and were cloned as *Kpn*I - *Sac*I fragments. Plasmid pRAB11-00585-myc encodes SAOUHSC_00585 with a C-terminal HA tag in pRAB11 and was constructed following amplification with primers 00585 cha fw and 00585 cha rv, digestion with *Bgl*II and *Eco*RI and cloning into similarly cut pRAB11. Plasmid pRMC2-ssaA-ha codes for SsaA with a C-terminal HA tag. It was constructed following amplification of SsaA using primers pRMC2-ssaA-bglII-for and pRMC2- ssaA-HA-rev-EcoRI, digestion with *Bgl*II and *Eco*RI and cloning into similarly cut pRMC2[12].

Plasmid pBAD18-tspA codes for the full length TspA. The encoding gene was amplified using primers tspA fl fw and tspA fl rv, digested with *Xba*I and *Sal*I and subsequently cloned into similarly cut pBAD18-Cm[9]. Plasmid pBAD18-tspA$_{CT}$ encodes for the last 251aa of TspA; the encoding DNA was amplified using primers tspA cp fw and tspA fl rv, digested with *Xba*I and *Sal*I and cloned into similarly cut pBAD18-Cm. Plasmid pBAD18-AmiAss-tspA$_{CT}$ codes for the *E. coli* AmiA signal sequence fused in-frame to the N-terminus of TspA$_{CT}$. This was constructed following separate amplification of the DNA encoding the first 36 amino acids of AmiA using primers AmiAss fw and AmiAss rv with *E. coli* MG1655 chromosomal DNA as template digestion with *Nhe*I and *Kpn*I and cloning into similarly cut pBAD18-Cm. The TspA$_{CT}$ coding sequence was then amplified using tspA cp fw (which lacks a start codon) and tspA fl rv, digested with *Xba*I and *Sal*I and cloned into similarly cut pBAD18-AmiAss. Plasmid pBAD18-AmiAss-tspA$_{CT}$ + tsaI codes for the AmiAss-

$TspA_{CT}$ fusion along with TsaI. The *tsaI* (*saouhsc_00585*) gene was amplified using primers tsaI fw and tsaI rv, digested with *Sal*I and *Sph*I and cloned into similarly cut pBAD18-AmiAss-$tspA_{CT}$.

Plasmid pSUPROM-TsaI produces TsaI constitutively from the *E. coli tat* promoter. The encoding gene was amplified using primers pSU-TsaI fw and pSU-TsaI rv, digested with *Bam*HI and *Xho*I and cloned into similarly-digested pSUPROM[10].

S. aureus strains were cultured in RPMI medium for proteomic analysis, as detailed below. For all other experiments *S. aureus* strains were grown in TSB medium at 37°C with vigorous agitation. Chloramphenicol was used 10 µg/ml final concentration for plasmid selection. Anhydrotetracycline (ATC) was added to *S. aureus* cultures at 1 µg/ml during allelic gene replacement. For induction of plasmid-encoded proteins, 500 ng/ml ATC was added to cultures at OD_{600} of 0.4, and cells were harvested at OD_{600} of 2.0. *E. coli* was grown aerobically in LB at 37°C, supplemented with ampicillin (100 µg/ml), kanamycin (50 µg/ml) or chloramphenicol (25 µg/ml) where appropriate. D-glucose and L-arabinose were used to control expression of cloned genes from the pBAD18-Cm vector[11]. For toxicity assays single colonies of MG1655 freshly transformed with the appropriate pBAD18-Cm construct growing on LB agar containing 0.2% D-glucose were picked and re-suspended in LB to an OD_{600} of 1.0. Serial dilutions to 10^{-5} were prepared and 5 µL of each spotted onto LB agar supplemented with 0.2% D-glucose, 0.02% L-arabinose or 0.2% L-arabinose and incubated overnight at 37°C. For growth curve measurements, overnight cultures of MG1655 harboring pBAD18-Cm constructs were sub-cultured to a starting OD_{600} of 0.1 (t=0) and incubated at 37°C and allowed to reach an OD_{600} of 0.5 before supplementation with 0.2% L-arabinose. Optical density readings at 600nm were taken for a 6 hour growth period, with readings collected every 2 hours. The number colony forming units was calculated at t=0 and every 2 hours post-induction with L-arabinose.

Serial dilutions to 10^{-6} were prepared and 100 µL of each plated onto LB agar containing 25 µg/ml chloramphenicol and incubated overnight at 37°C.

Preparation of culture supernatants for proteomic analysis. *S. aureus* strains were grown overnight in 2 mL TSB after which cells were harvested, washed three times with 10 mL of RPMI medium, resuspended in 2 mL of RPMI and used to inoculate 200 mL RPMI in 2 L baffled flasks. Cultures were grown at 37°C with vigorous agitation until an OD_{600} of 1.0 was reached, after which cultures were cooled to 4°C, cells pelleted and supernatant proteins precipitated with 6% trichloroacetic acid (TCA) on ice overnight. The precipitated protein samples were harvested by centrifugation (15 min at 18000 *g*) re-suspended in 80% acetone (-20°C) and washed twice with 80% acetone. Pellets were air dried at room temperature and transferred to the mass-spectrometry facility for proteomic analysis.

Mass spectrometry data analysis and label-free quantitation. Sample preparation and mass spectrometry analysis was performed similar to previously described work[13-16]. Precipitated proteins were re-dissolved in 1% sodium 3-[(2-methyl-2-undecyl-1,3-dioxolan-4-yl)methoxy]-1-propanesulfonate (commercially available as RapiGest, Waters), 50 mM Tris-HCl pH 8.0, 1 mM TCEP. Cysteines were alkylated by addition of 20 mM Iodoacetamide and incubation for 20 min at 25°C in the dark and the reaction quenched by addition of 20 mM DTT. Samples were diluted to 0.1% Rapigest with 50 mM Tris-HCl pH 8.0 and Trypsin (sequencing grade, Promega) was added at a 1:50 ratio. Proteins were digested overnight at 37°C under constant shaking.

Samples from four biological replicates (0.5 µg of digest for the secretome analyses) were injected in an interleaved manner onto a 2 cm x 100 µm trap column and separated on a 50 cm x 75 µm EasySpray Pepmap C18 reversed-phase column (Thermo Fisher

Scientific) on a Dionex 3000 Ultimate RSLC. Peptides were eluted by a linear 3-hour gradient of 95% A/5% B to 35% B (A: H_2O, 0.1% Formic acid (FA); B: 80% ACN, 0.08% FA) at 300 nl/min into a LTQ Orbitrap Velos (Thermo-Fisher Scientific). Data was acquired using a data-dependent "top 20" method, dynamically choosing the most abundant precursor ions from the survey scan (400-1600 Th, 60,000 resolution, AGC target value 10^6). Precursors above the threshold of 2000 counts were isolated within a 2 Th window and fragmented by CID in the LTQ Velos using normalized collision energy of 35 and an activation time of 10 ms. Dynamic exclusion was defined by a list size of 500 features and exclusion duration of 60 s. Lock mass was used and set to 445.120025 for ions of polydimethylcyclosiloxane (PCM).

Label-free quantitation was performed using MaxQuant 1.5.7.4[17]. Data were searched against the Uniprot database of *S. aureus* NCTC8325 (downloaded on 29.03.17) containing 2,889 sequences and a list of common contaminants in proteomics experiments using the following settings: enzyme Trypsin/P, allowing for 2 missed cleavage, fixed modifications were carbamidomethyl (C), variable modifications were set to Acetyl (Protein N-term), Deamidation (NQ) and Oxidation (M). MS/MS tolerance was set to 0.5 Da, precursor tolerance was set to 6 ppm. Peptide and Protein FDR was set to 0.01, minimal peptide length was 7, and one unique peptide was required. Re-quantify and retention time alignment (2 min) were enabled. If no intensities were detected in one condition and the other condition had intensities in at least in 3 out of 4 replicates, values were imputed in Perseus v1.5.1.1 using default parameters[18]. A student's t-test (two-tailed, homoscedastic) was performed on the LFQ intensities and only proteins with $p<0.05$ and a fold-change >2-fold were considered significant.

Bacterial membrane potential detection. To assess bacterial membrane potential, the method of Miyata *et al.*[19] was adapted, using the BacLight bacterial membrane potential kit (Invitrogen). In brief, overnight cultures of *E. coli* MG1655 harboring pBAD18-Cm derivatives were sub-cultured into LB medium containing appropriate antibiotics to an OD_{600} of 0.1 and cultured aerobically to OD_{600} of 0.5, before supplementation with 0.2% L-arabinose. Cells were cultured for a further hour then diluted to 1 x 10^6 cells per ml in sterile PBS, and a 1ml aliquot of bacterial suspension was added to each flow cytometry tube. For the depolarized control, 25 μL of 500 μM carbonyl cyanide m-chlorophenyl hydrazone (CCCP), provided in the BacLight bacterial membrane potential kit, was added. Next, 3 μL of 3mM $DiOC_2(3)$ was added to each sample, which was mixed and incubated at room temperature for 30 minutes. Cells were subsequently sorted using an LSRFortessa cell analyzer (BD Biosciences, San Jose, CA). The $DiOC_2(3)$ dye was excited at 488 nm, with fluorescent emissions detected using Alexa488 (Ex 488nm, Em 530/30 nm) and Alexa568 (Ex 561 nm, Em 610/20 nm). In total, 20000 events were collected for each sample, with forward- and side-scatter parameters used to gate the bacteria. The forward scatter, side scatter, and fluorescence were collected with logarithmic signal amplification. The gated populations were then analyzed with FlowJo software by generating a dot plot of red versus green fluorescence.

To assess changes in membrane potential and permeabilization, the same *E. coli* strains were grown as described above. An 'uninduced' sample of *E. coli* harboring each of pBAD18-Cm (empty), pAmiAss-$TspA_{CT}$ and pAmiAss-$TspA_{CT}$-TsaI was collected and adjusted to OD_{600} of 0.2 before the addition of 2 μM $DiSC_3(5)$ and 200 nM Sytox Green. A permeabilized control sample of cells harboring empty vector and containing 10 μg/ml Polymyxin B was also prepared and supplemented with both dyes. All samples were then incubated at 37°C for 5 minutes before being analyzed by microscopy. To induce protein production from the pBAD18-Cm vector, cell suspensions were adjusted to OD_{600} of 0.2

and supplemented with 0.2% L-arabinose for the indicated period of time (10-60 min) before incubating with $DiSC_3(5)$ and Sytox Green, as above. Imaging of $DiSC_3(5)$ and Sytox Green stained cells was carried out Nikon Eclipse Ti equipped with Sutter Instrument Lambda LS light source, Nikon Plan Apo 100×/1.40 NA Oil Ph3 objective, and Photometrics Prime sCMOS, and Cy5 and GFP filters, respectively. The images were captured using Metamorph 7.7 (Molecular Devices) and analyzed using ImageJ. For the analysis, the phase contrast images acquired in parallel to the fluorescence images were used to identify cells as regions of interest, for which average $DiSC_3(5)$ and Sytox Green fluorescence intensity was measured from the corresponding background-subtracted fluorescence images. Data was then plotted as a scatter plot for $DiSC_3(5)$ and Sytox Green fluorescence with each point representing an individual cell.

Mouse pneumonia model. *S. aureus* RN6390 (WT) or the isogenic Δ*essC* strain were subcultured at 1:100 dilution from an overnight culture into fresh TSB medium. Cells were grown at 37°C with shaking until an OD_{600} of 0.5 was reached, before harvesting and washing three times in 1x PBS. Cells were finally resuspended in 1 x PBS to 1.2 x 10^{10} cfu/ml. Female 10-12 week old C57/B6 J mice were purchased from Charles River U.K.. Mice were acclimatized for a 10 day period prior to starting the experiment. Mice were randomized to cages and treatment groups, and analyses were performed blind. Mice were anaesthetized (gaseous isofluorane) and infected intranasally with 25 µL of the bacterial suspension to give a final infected dose of 3 x 10^8 cfu per mouse. At 24 hours post infection the lungs and livers were harvested and the bacterial load determined by plating serial dilutions of tissue homogenates. Experiments were approved by the University of Dundee Welfare and Ethics Committee and performed under Project Licence

authority in compliance with UK Home Office Animals (Scientific Procedures) Act 1986 guidelines (Project licence: PEA2606D2).

Quantitative reverse transcription polymerase chain reaction (qRT-PCR). For RNA was harvested from 10 snap-frozen zebrafish larvae infected with *S. aureus* at 6 hpi using the RNeasy Mini Kit (Qiagen) as per manufacturer's instructions. RNA was reverse transcribed into cDNA using QuantiTect Reverse Transcription Kit (Qiagen) as per manufacturer's instructions with 500 ng of RNA. Quantitative RT-PCR reactions were performed on four biological replicates, each with two technical replicates. For qRT-PCR reactions, 50 ng of cDNA was used per reaction with SYBR Green Reaction Mix (Thermo Fisher Scientific) on a Rotor GeneQ (Qiagen) thermocycler. Oligonucleotides for *il-1b*, *cxcl8* and *eef1a1a* are listed in *SI Appendix*, Table S3. Quantities of cDNA were normalised using the housekeeping gene *eef1a1a* and the 2-ΔΔCT method was used for analysis[20].

Imaging of *S. aureus* – leukocyte interactions *in vivo*. To observe *S. aureus* – leukocyte interactions, Tg(*lyz*::dsRed)^{nz50} and Tg(*mpeg1*::Gal4-FF)^{gl25}/Tg(*UAS-E1b*::*nfsB*.mCherry)^{c264} zebrafish larvae were infected with *S. aureus* strains chromosomally labelled with GFP. To follow neutrophil and macrophage recruitment, infected larvae were anaesthetised with 200 µg/ml Tricaine and the HBV imaged at 0, 3, and 6 hpi by fluorescence stereomicroscopy and multiple position z stacks of up to 400 µm were acquired using a Leica M205FA stereomicroscope with a 10x (NA 0.5) dry objective. Neutrophil and macrophage quantifications were performed manually throughout the individual z stacks using Fiji – ImageJ (ver 1.0).

Statistical analysis. For statistical analysis GraphPad Prism 7.0 software was used. In survival assays statistical analysis was done using a Log rank (Mantel-Cox) test. To analyse bacterial kinetics, CFU counts were Log10 transformed and the significance between two independent groups was determined by an unpaired *t* test. When more than two groups were compared, significance was determined by using one-way ANOVA with Sidak's comparison. Gene expression levels were quantified on Log2 data and significance was determined by using one-way ANOVA with Sidak's Multiple Comparison test. For leukocyte cell counts analysis (non-parametric data), significance between multiple selected groups was determined using Kruskal-Wallis test with Dunn's correction. Significance is indicated ns, non-significant, *, $p \leq 0.05$; **, $p \leq 0.01$; ***, $p \leq 0.001$; ****, $p \leq 0.0001$.

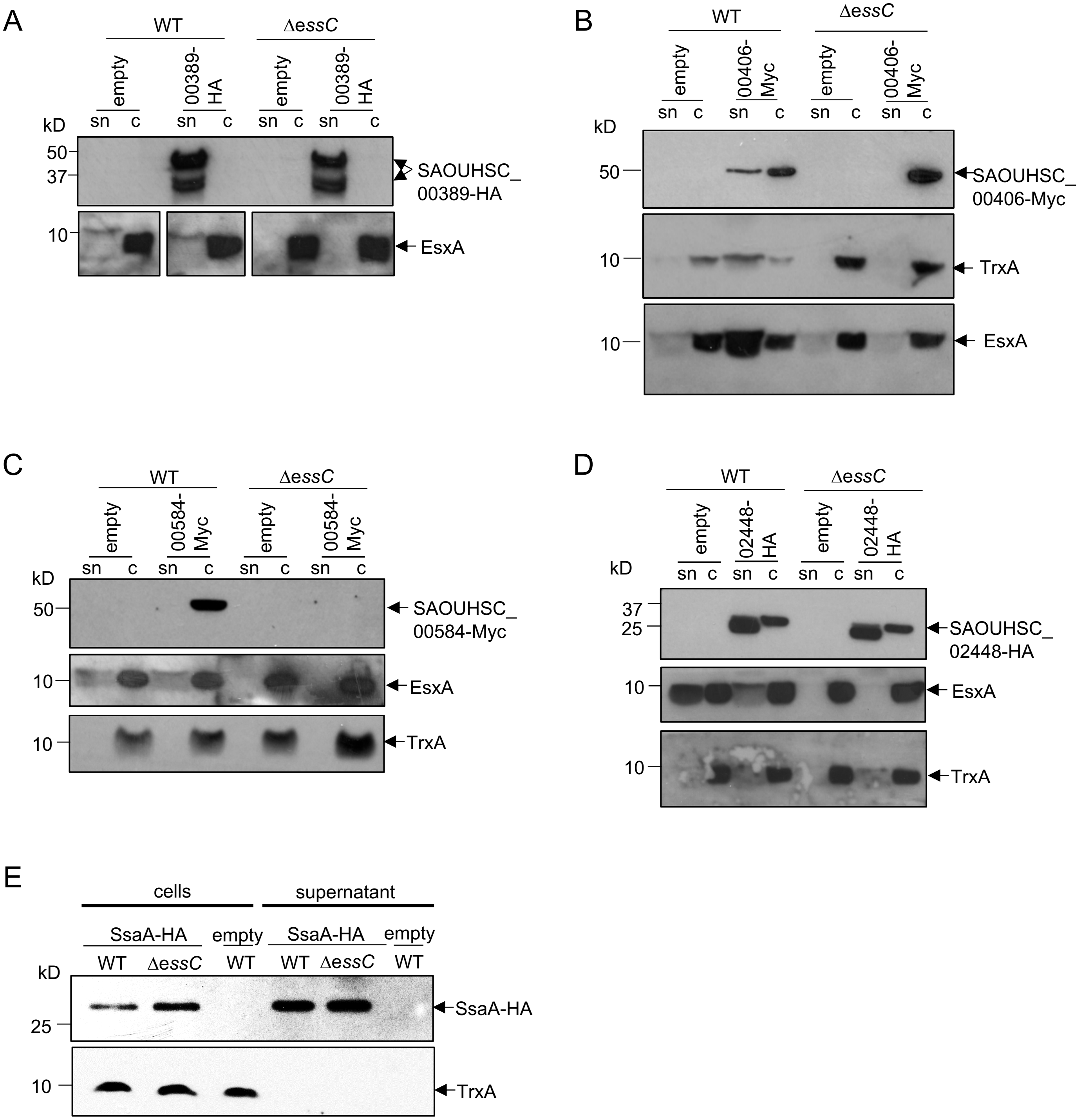

Figure S1. HA-tagged variants of SAOUHSC_00389, SAOUHSC_02448 and SsaA are not secreted by the T7SS. The wild type RN6390 and Δ*essC* mutant carrying A-D pRAB11 (empty) or pRAB11 encoding A. C-terminally HA-tagged SAOUHSC_00389; B. C-terminally Myc-tagged SAOUHSC_00406; C. C-terminally Myc-tagged tagged SAOUHSC_00584; or D. C-terminally HA-tagged SAOUHSC_02448, or E. pRMC2 (empty) or encoding C-terminally HA-tagged SsaA were cultured in TSB medium and supplemented with 200ng/ml ATc at OD_{600} of 0.5. When cultures reached OD_{600} of 2.0, samples were withdrawn and separated into culture supernatant (sn) and cellular (c) fractions. Samples were separated on 12% bis-Tris gels and immunoblotted with anti-HA, anti-Myc, anti-EsxA or anti-TrxA antibodies, as indicated.

MSIDMYLDRSRNQASSVGNLSQTMNSNYDALEKAITQFINDDALKGKAYTSAKQFFSTVLIPLSTSMK
TLSDLTKQACDNFVSRYTSEVDSISLKESELEEDIRSLSQQITRYENLNNNLKKHASDNQQAISSNQQ
IIRTLGQQKHELEEKLRKLREFNQKSPEIFKEVEEFQKIVQQGLTQAQNFWNFSTNQFNIPSGKELDW
AKASHEKYLKVAMGKIEHKAEKETLNKADFAVIKAYAKEHPEDDIPKSILKYINDNKDSIKRDIGLDI
TSTLLEQDGINASKFGVFINTAGGVKGPAGPNSFVEVKRTSGNVFIENGSKFAKGGKYLGKGVAGVGF
GIGMYDDLANDDKTFGEALSHNGMTLAAGSAGTAVGAGLATFVLGSNPVGWVILAGLAMSTVFALGTD
LIYQNNIFGLKDKVDWVGHKIDNSIDVVKKTTEKSMDSVGNAVSEAKNIISNHINPMKWAW

Figure S2. Peptide coverage of TspA from proteomic analysis. Regions of TspA coloured orange were detected by mass spectroscopy analysis.

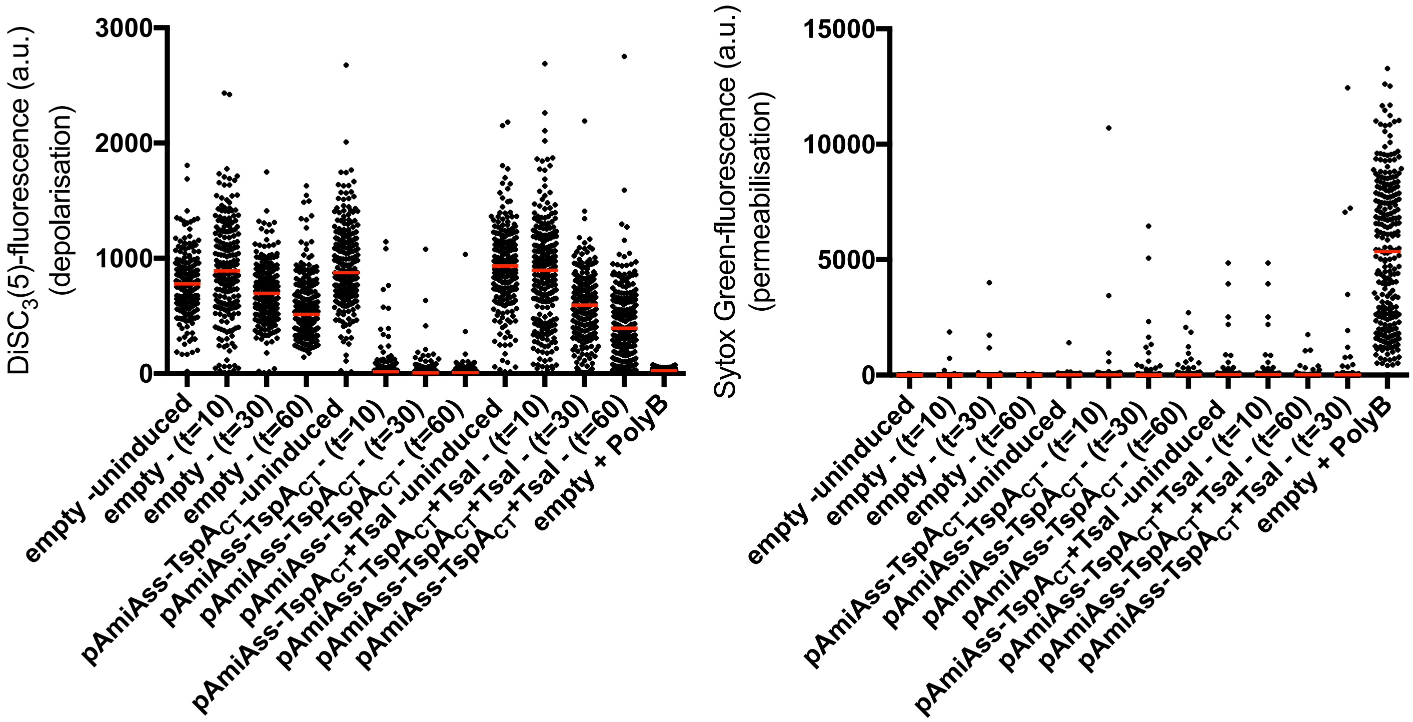

Figure S3. Rapid membrane depolarization by the C-terminal domain of TspA. Fluorescence intensity changes in DiSC$_3$(5) (left) and Sytox Green (right) in uninduced cells harbouring pBAD18-Cm (empty), pBAD18-AmiAss-TspA$_{CT}$ or pBAD18-AmiAss-TspA$_{CT}$/TsaI and upon induction with 0.2% L-arabinose. Cells were then induced for 10, 30 and 60 minutes before samples removed and cells incubated with DiSC$_3$(5) and Sytox Green. Fluorescence intensity of DiSC$_3$(5) and Sytox Green also measured upon addition of Polymixin B to empty vector as a control for membrane depolarization and permeabilization.

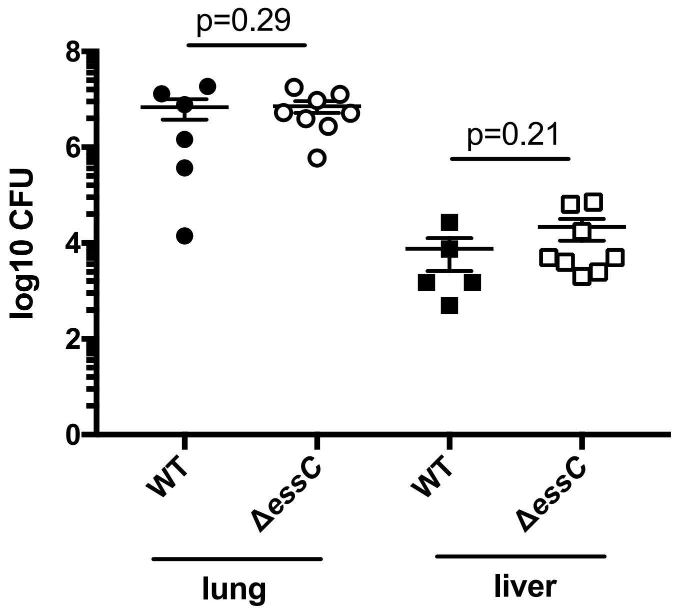

Figure S4. No difference in bacterial burden between wild type and *essC* mutant strains in a 24 hour murine pneumonia infection model. Previous reports have indicated that 2-4 x 10^8 cfu of strain Newman was a suitable infectious dose, and that bacterial proliferation in lung tissue could be observed after 24 hours[21]. We found that at a dose of 8 x 10^7 – 2 x 10^8 of strain RN6390, the mice were asymptomatic and had almost completely cleared the bacteria from lung tissue after 48 hours, whereas a dose of 8 x 10^8 – 2 x 10^9 was lethal to all mice within 12 hours. At a dose of 3 x 10^8, the mice developed symptoms which resolved within 12 hours, and therefore using this dosage we sought to test whether there was a difference in bacterial proliferation and dissemination dependent on the T7SS. However, after 24 hours of infection with 3 x 10^8 cfu of RN6390 or a cognate Δ*essC* strain, counts recovered from the lungs and livers of mice infected with the Δ*essC* strain were not significantly different than those from mice infected with the wild type. Here in Figure S4, female 10-12 week old C57/B6 mice were challenged with 3 x 10^8 cfu/ml of RN6390 (WT) or the isogenic Δ*essC* strain. Bacterial load was determined in liver and lungs 24 hours after infection. Mean ± SEM (horizontal bars) is shown. Significance testing performed by unpaired *t* test.

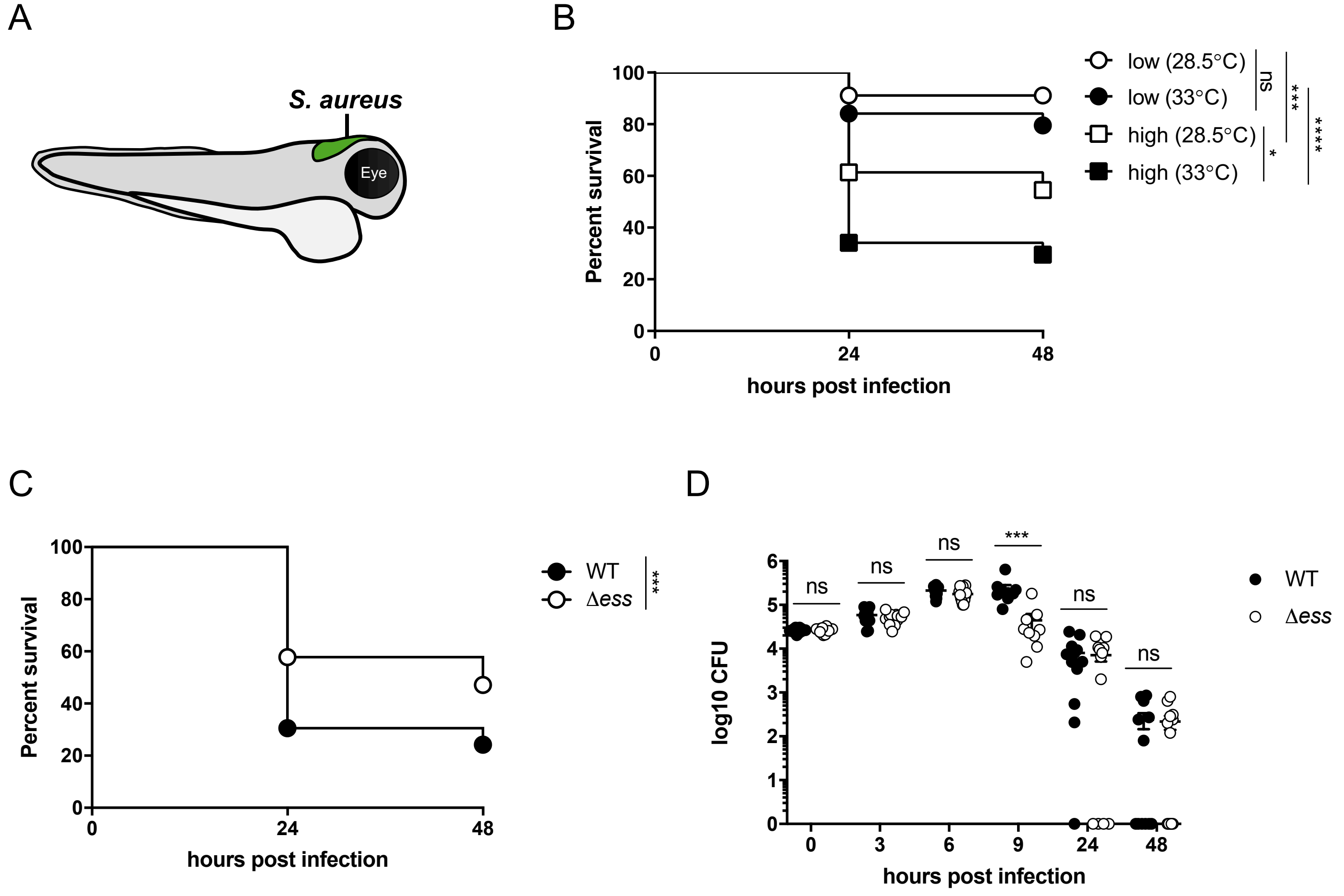

Figure S5. Developing a zebrafish model to assess T7SS activity *in vivo*. A. Schematic of zebrafish larvae showing the site of *S. aureus* injection into the hindbrain ventricle. B. Survival curves of *lyz*:dsRed zebrafish larvae injected with wild type RN6390 chromosomally tagged with GFP. Zebrafish were injected at 3 dpf with a low (~7 x 10^3 cfu), medium (~1.4 x 10^4 cfu) or high (~2 x 10^4 cfu) dose of RN6390-gfp, incubated at 28.5°C or 33°C and monitored for 48 hpi. Data are pooled from two independent experiments (*n*=22-25 larvae per experiment). Results are plotted as a Kaplan- Meier survival curve and the *p* value between conditions was determined by log-rank Mantel-Cox test. C. Survival curves of *lyz*:dsRed larvae infected in the hindbrain 3 dpf with RN6390-gfp or RN6390 Δ*ess*-gfp at a dose of ~2 x 10^4 cfu and incubated at 33°C for 48 hpi. Data are pooled from three independent experiments (*n*=28-50 larvae per experiment. Results are plotted as a Kaplan-Meier survival curve and the *p* value between conditions was determined by log-rank Mantel-Cox test. D. Enumeration of recovered bacteria at 0, 3, 6, 9, 24 or 48 hpi from zebrafish larvae infected with RN6390-gfp or RN6390 Δ*ess*-gfp. Pooled data from 3 independent experiments. Circles represent individual larva, and only larvae having survived the infection were included. Mean ± SEM also shown (horizontal bars). Significance was tested using an unpaired *t* test. *p<0.05 **p<0.01, *** p<0.001, **** p<0.0001, ns, not significant.

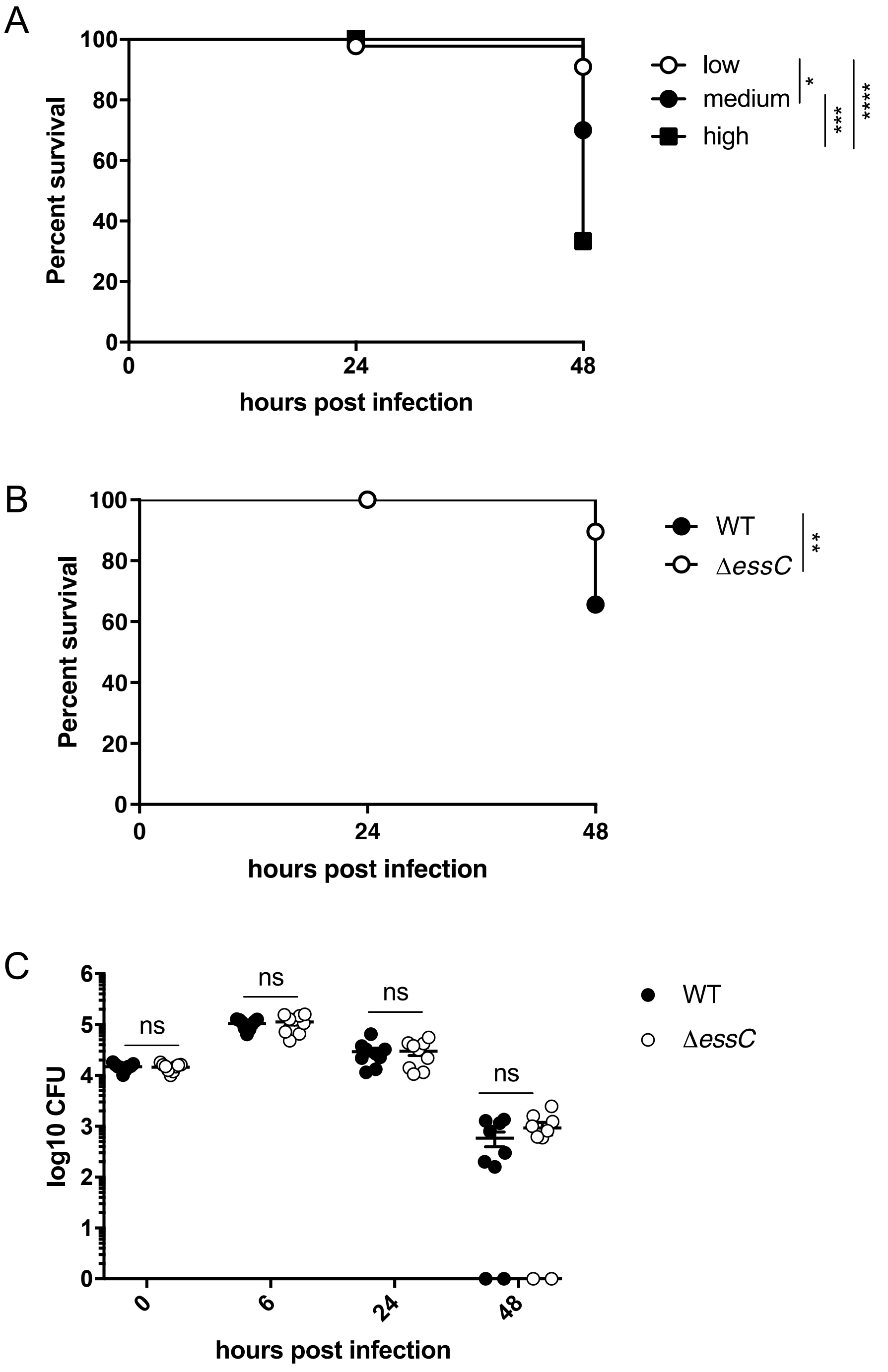

Figure S6. *S. aureus* COL also exhibits dose and T7SS-dependent zebrafish mortality. A. Survival curves of WT zebrafish larvae injected with wild type COL chromosomally tagged with mCherry. Zebrafish were injected at 3 dpf with a low (~7 x 10^3 cfu), medium (~1.5 x 10^4 cfu) or high dose (~2 x 10^4 cfu) of COL-mCherry, incubated at 33°C and monitored for 48 hpi. Data are pooled from three independent experiments. Results are plotted as a Kaplan-Meier survival curve and the *p* value between conditions was determined by log-rank Mantel-Cox test. B. Survival curves of zebrafish larvae injected in the hindbrain 3 dpf with COL-mCherry (WT) or COL Δ*essC*-mCherry at a dose of ~1.6 x 10^4 cfu and incubated at 33°C for 48 hpi. Data are pooled from three independent experiments (*n*=23-30 larvae per experiment). Results are plotted as Kaplan-Meier survival curves and the *p* value between conditions was determined by the log-rank Mantel Cox test. C. Enumeration of recovered bacteria at 0, 6, 24 or 48 hpi from zebrafish larvae infected with COL-mCherry (WT) or COL Δ*essC*-mCherry. Circles represent individual larvae and data pooled data from 3 independent experiments. Mean ± SEM also shown (horizontal bars). Significance was tested using an unpaired *t* test. *$p<0.05$ **$p<0.01$, *** $p<0.001$, **** $p<0.0001$, ns, not significant.

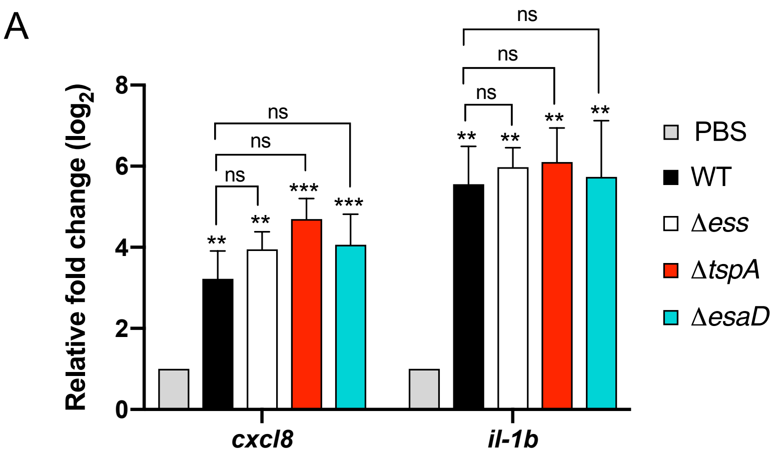

Figure S7. *S. aureus* infection elicits a strong inflammatory response independent of the T7SS. A. *S. aureus* RN6390-gfp, RN6390 Δ*ess*-gfp, Δ*tspA*-gfp or RN6390 Δ*esaD*-gfp were injected in 3 dpf zebrafish larvae at a dose of ~2 x 10^4 CFU and incubated at 33°C. Expression of *cxcl8* and *il-1b* was determined at 6 hpi when the bacterial burden between among strains was similar. Mean relative *cxcl8* and *il-1b* gene expression levels (qRT-PCR) were quantified and values were normalised to the PBS-injected larvae. Therefore, the *p* value (indicated above the bars) represents the statistical significance of the indicated *S. aureus* strains in comparison to the PBS-injected larvae. Pooled data from 4 independent experiments where 10 larvae were sacrificed after 6 hpi. Error bars represent mean with SEM (horizontal bars). Significance was performed using a one-way ANOVA with Sidak's correction. *p<0.05 **p<0.01, *** p<0.001, **** p<0.0001, ns, not significant.

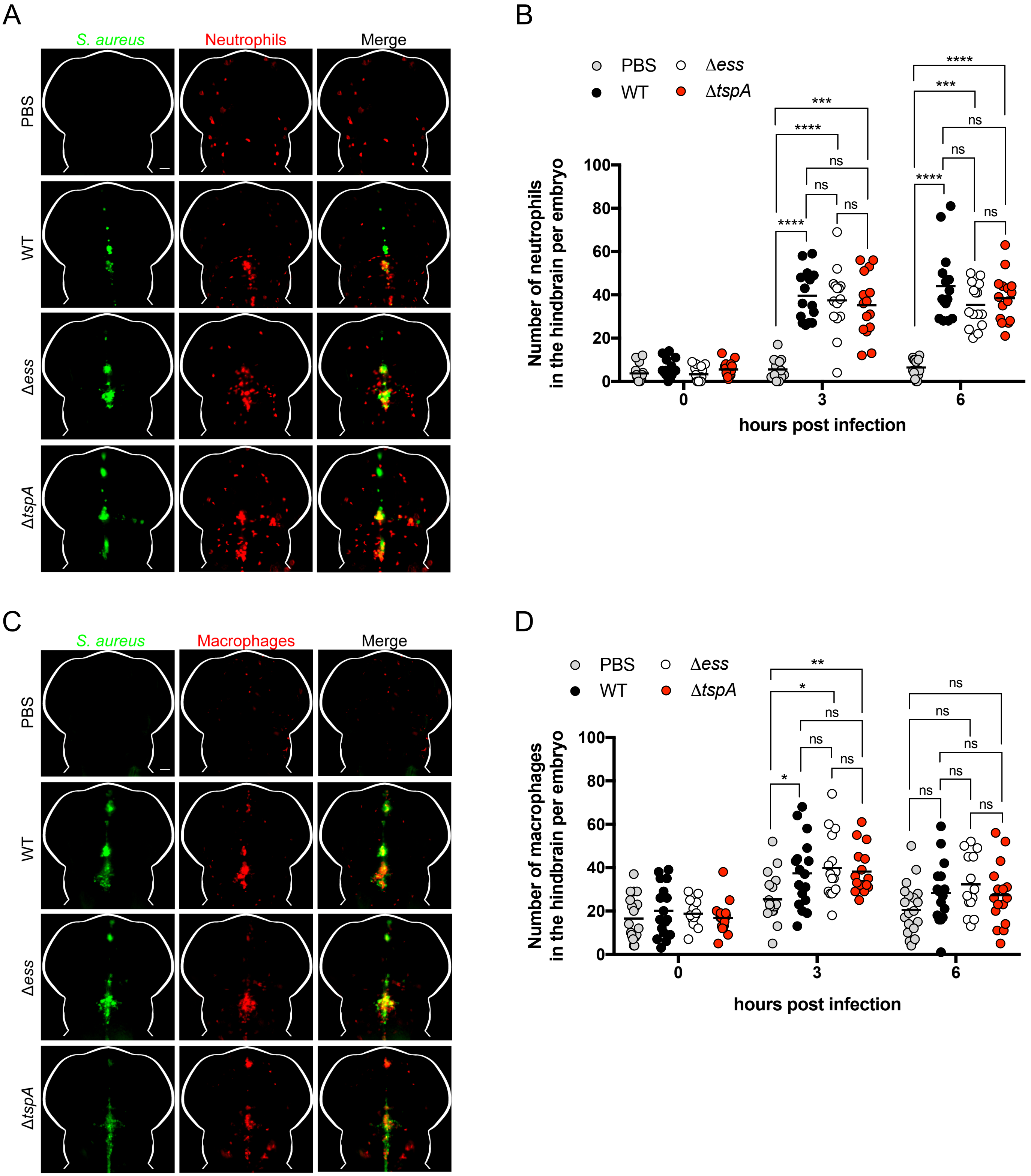

Figure S8. T7SS independent leukocyte recruitment to *S. aureus* infection. A+B. Neutrophils were imaged and counted in the whole hindbrain for Tg(*lyz*::dsRed) larvae and C+D. Macrophages were imaged and counted in the whole hindbrain for Tg(*mpeg1*::G/U::mCherry) larvae injected with PBS, RN6390-gfp, RN6390 Δ*ess*-gfp or RN6390 Δ*tspA*-gfp. Larvae were imaged using a fluorescent stereomicroscope at 0, 3 and 6 hpi and data obtained from three independent experiments with 5-6 larvae imaged per strain. Data points represent an individual larvae with the geometric mean. Significance testing performed using Kruskal-Wallis test with Dunn's correction. *$p<0.05$ **$p<0.01$, *** $p<0.001$, **** $p<0.0001$, ns, not significant. Representative images of a single z stack of neutrophil or macrophage recruitment at 6 hpi is shown in A+C, respectively. Leukocytes are labelled in red and *S. aureus* in green, with overlay in yellow. Scale bar = 50 μm.

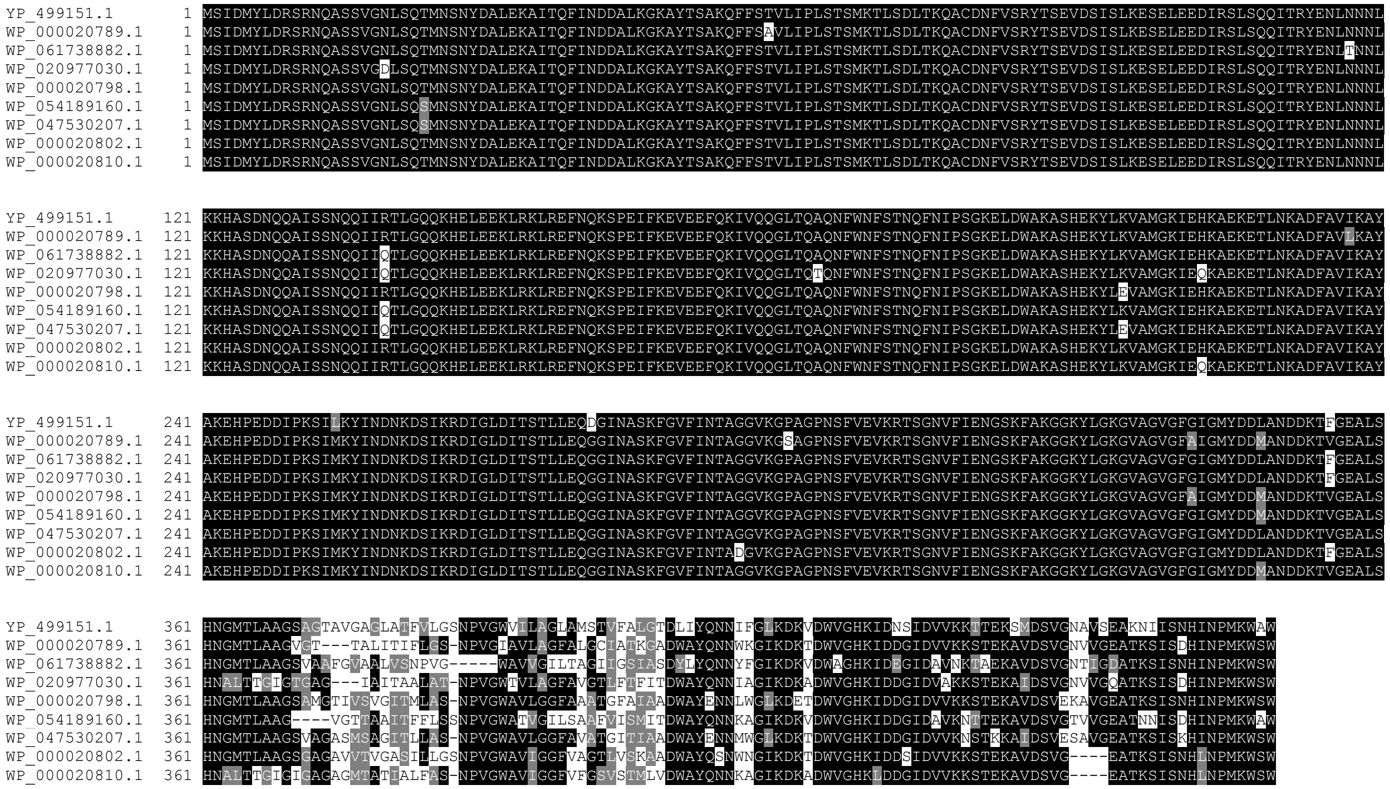

Figure S9. TspA proteins encoded by *S. aureus* strains show variability within the channel-forming domain. A selection of TspA protein sequences encoded by *S. aureus* strains were extracted from NCBI (ncbi.nlm.nih.gov/), aligned using ClustalW (http://www.ch.embnet.org/software/ClustalW.html) and shaded with Boxshade (http://www.ch.embnet.org/software/BOX_form.html).

SI References

1. Novick, R.P. *et al.* Synthesis of staphylococcal virulence factors is controlled by a regulatory RNA molecule. *EMBO J* **12**, 3967-3975 (1993).

2. Kneuper, H. *et al.* Heterogeneity in ess transcriptional organization and variable contribution of the Ess/Type VII protein secretion system to virulence across closely related *Staphylocccus aureus* strains. *Mol Microbiol* **93**, 928-943 (2014).

3. Cao, Z., Casabona, M.G., Kneuper, H., Chalmers, J.D. & Palmer, T. The type VII secretion system of *Staphylococcus aureus* secretes a nuclease toxin that targets competitor bacteria. *Nat Microbiol* **2**, 16183 (2016).

4. Dyke, K.G., Jevons, M.P. & Parker, M.T. Penicillinase production and intrinsic resistance to penicillins in *Staphylococcus aures*. *Lancet* **1**, 835-838 (1966).

5. Blattner, F.R. *et al.* The complete genome sequence of *Escherichia coli* K-12. *Science (New York, N.Y* **277**, 1453-1462 (1997).

6. Grahl, S. Tat signal peptide recognition during protein maturation and export. doctoral degree thesis, University of Dundee, Dundee, 2011.

7. Monk, I.R., Shah, I.M., Xu, M., Tan, M.W. & Foster, T.J. Transforming the untransformable: application of direct transformation to manipulate genetically *Staphylococcus aureus* and *Staphylococcus epidermidis*. *MBio* **3** (2012).

8. de Jong, N.W., van der Horst, T., van Strijp, J.A. & Nijland, R. Fluorescent reporters for markerless genomic integration in *Staphylococcus aureus*. *Sci Rep* **7**, 43889 (2017).

9. Guzman, L.M., Belin, D., Carson, M.J. & Beckwith, J. Tight regulation, modulation, and high-level expression by vectors containing the arabinose P_{BAD} promoter. *J Bacteriol* **177**, 4121-4130 (1995).

10. Jack, R.L. *et al.* Coordinating assembly and export of complex bacterial proteins. *EMBO J* **23**, 3962-3972 (2004).

11. Helle, L. *et al.* Vectors for improved Tet repressor-dependent gradual gene induction or silencing in *Staphylococcus aureus*. *Microbiology* **157**, 3314-3323 (2011).

12. Corrigan, R.M. & Foster, T.J. An improved tetracycline-inducible expression vector for *Staphylococcus aureus*. *Plasmid* **61**, 126-129 (2009).

13. Fritsch, M.J. *et al.* Proteomic identification of novel secreted antibacterial toxins of the *Serratia marcescens* type VI secretion system. *Mol Cell Proteomics* **12**, 2735-2749 (2013).

14. Hamilton, J.J. *et al.* A holin and an endopeptidase are essential for chitinolytic protein secretion in *Serratia marcescens*. *J Cell Biol* **207**, 615-626 (2014).

15. Cianfanelli, F.R. *et al.* VgrG and PAAR Proteins Define Distinct Versions of a Functional Type VI Secretion System. *PLoS Pathog* **12**, e1005735 (2016).

16. Trunk, K. *et al.* The type VI secretion system deploys antifungal effectors against microbial competitors. *Nat Microbiol* **3**, 920-931 (2018).

17. Cox, J. & Mann, M. MaxQuant enables high peptide identification rates, individualized p.p.b.-range mass accuracies and proteome-wide protein quantification. *Nat Biotechnol* **26**, 1367-1372 (2008).

18. Tyanova, S. *et al.* The Perseus computational platform for comprehensive analysis of (prote)omics data. *Nat. Methods* **13**, 731-740 (2016).

19. Miyata, S.T., Unterweger, D., Rudko, S.P. & Pukatzki, S. Dual expression profile of type VI secretion system immunity genes protects pandemic *Vibrio cholerae*. *PLoS Pathog* **9**, e1003752 (2013).

20. Livak, K.J. & Schmittgen, T.D. Analysis of relative gene expression data using real-time quantitative PCR and the 2-ΔΔCT method. *Methods* **25,** 402–408 (2001).

21. Bubeck Wardenburg, J., Patel, R.J. & Schneewind, O. Surface proteins and exotoxins are required for the pathogenesis of *Staphylococcus aureus* pneumonia. *Infect Immun* **75**, 1040-1044 (2007).
